# Numerical comparison of nonlinear thermal radiation and chemically reactive bio-convection flow of Casson-Carreau nano-liquid with gyro-tactic microorganisms: Lie group theoretic approach

**DOI:** 10.1016/j.heliyon.2024.e29568

**Published:** 2024-04-16

**Authors:** Musharafa Saleem, Majid Hussain

**Affiliations:** a Department of Mathematics, University of Management and Technology, Sialkot Campus 51310, Pakistan; b Department of Natural Sciences and Humanities, University of Engineering and Technology, Lahore 54890, Pakistan

**Keywords:** Casson-Carreau nanofluid (CCNF), Buongiorno nanofluid model, Magnetohydrodynamics (MHD), Stefan blowing, Bio-convection, Lie scaling method

## Abstract

The current research presents a mathematical model to study the flow of a non-Newtonian magnetohydrodynamics (MHD) Casson-Carreau nanofluid (CCNF) over a stretching porous surface, considering mass and heat transport rates with Stefan blowing, non-linear thermal radiation, heat source-sink, chemical reaction, thermophoretic and Brownian motions, convective heating, Joule heating, motile microorganisms, and bio-convection. The presence of microorganisms is utilized to control the suspension of nanomaterials within the nanofluid. The current flow model has been rendered by the boundary layer approximation and we get the highly nonlinear partial differential equations (PDEs). These nonlinear PDEs are simplified by the novel Lie group theoretic method. The one-parameter Lie scaling method simplified the PDEs and convert it into the ordinary differential equations (ODEs). Numerical solutions for these ODEs are obtained using the bvp4c scheme built-in function in MATLAB, ensuring reliable outcomes for temperature, velocity, concentration, and motile microorganism density profiles. The numerical results are presented through graphs and compared with available data, showing good agreement. These numerical outcomes reveal several important flow characteristics. Rates of change for *Nr* are 0.0007 and 0.0005, and for Ω, they are −0.0754 and −0.0536, respectively. Similarly, the rate of change for *Rb* in both models is −0.002 and −0.0002. Analysis shows a positive impact of the bioconvection Rayleigh number in both models, notably higher for the Casson fluid compared to the Carreau fluid model. Buoyancy ratio parameter exhibits consistent rates of change, while the reduction in impact is more pronounced for the Casson fluid model in the case of the mixed bioconvection parameter. The mixed bio-convection parameter reduces momentum velocity for both Casson and Carreau fluids, whereas the Darcy parameter boosts fluid velocity. As the Newtonian heating parameter increases, the temperature velocity distribution of both fluids also increases. The concentration profile of both Casson-Carreau fluid phases declines as the heat source-sink parameter and Schmidt number increase. Microbial velocity shows a decrease with increasing *R* values, whereas the opposite trend is observed for the Peclet number *Pe*.

**Nomenclature**

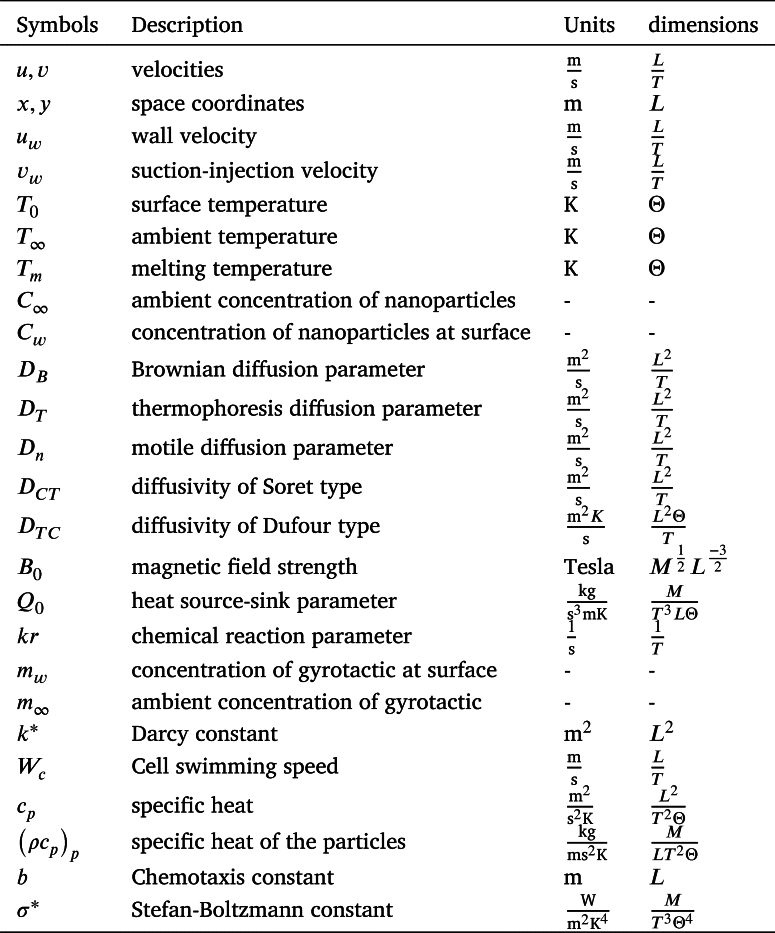



**Greeks**

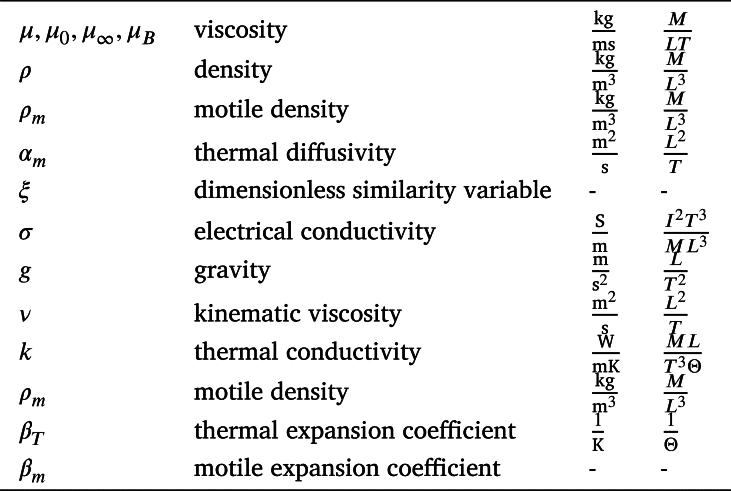



**Dimensionless numbers**

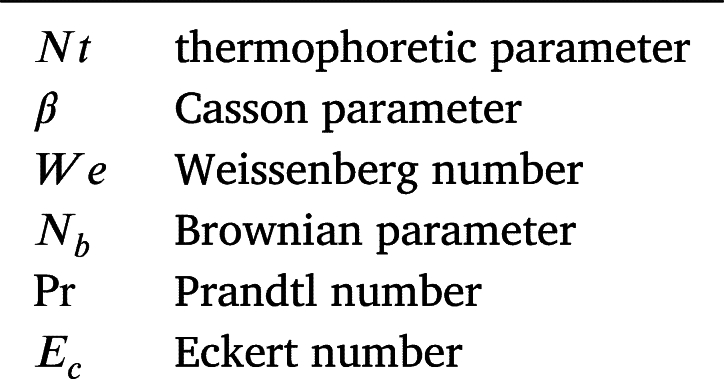


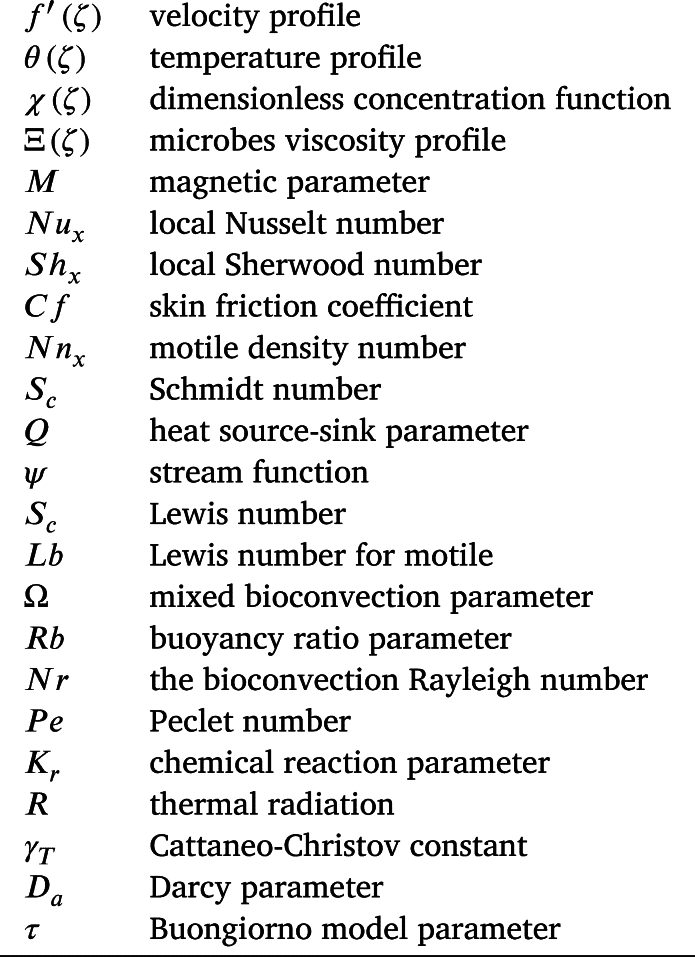



**Subscripts**

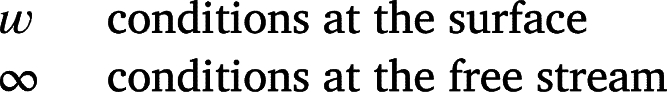



## 1 Introduction

Researchers are increasingly directing their attention towards non-Newtonian fluids due to their diverse properties, which hold significant potential across technical, commercial, and medicinal fields. These fluids, ranging from artificial lubricants to physiological liquids like blood, exhibit a variety of behaviors, presenting challenges in mathematical formulation as they defy the Navier–Stokes expressions. Consequently, scientists have developed several models to tackle this issue, with the Casson and Carreau models being the most prominent. Applications of such fluids span across industries, from paints and mining slurries to ice cream and cleansers. The flow of non-Newtonian fluids over a stretching sheet within the boundary layer approximation has attracted considerable interest due to its extensive applications across manufacturing, scientific, and engineering domains. Examples range from producing glass fiber and coolants to designing solar aircraft, ventilation systems, transportation, and water treatment processes. Non-Newtonian fluids exhibit varying viscosity or stress-flow characteristics over time or space. Some fluids undergo shear-thinning when subjected to external forces like agitation, while others may thicken with deliberate movement. However, they typically revert to their original state once the applied stress is removed. This dynamic behavior presents challenges and opportunities in optimizing processes across industries, necessitating a thorough understanding and accurate modeling of fluid flow for enhanced performance and efficiency.

In 1995, Casson introduced a fluid model pertinent to non-Newtonian liquids. This structural model is rooted in the interaction between liquid and solid phases, revealing a distinct yield stress. When the yield stress exceeds the applied shear force, the liquid behaves akin to a solid. Conversely, if the yield stress is surpassed by the shear stress, the liquid begins to flow. Concentrated juices, jelly, human blood, tomato sauce, and honey exemplify Casson liquids. The Casson fluid model finds diverse applications in areas such as cancer therapy, fibrinogen research, and blood cell dynamics. As a result, numerous scientists have been inspired by Casson liquids and have characterized their attributes, considering various physical aspects. Hayat et al. [1] conducted a study on the flow of magnetic Casson fluid over a stretching surface, considering the influence of thermal effects such as Soret and Dufour effects. Another investigation by Shehzad et al. [2] incorporated chemical reaction into the boundary layer approximation for the Casson magnetohydrodynamic model, while also examining suction and injection phenomena. In a separate study, Hayat et al. [3] explored mixed convection effects on a convectively heated stretching surface, integrating chemical reaction and heat source-sink effects. Rauf et al. [4] contributed to the literature by introducing a three-dimensional boundary layer approximation for the Casson nanofluid model, investigating mixed convection and convective conditions.

The Carreau model stands as a significant category among rate-type non-Newtonian fluids, offering a pivotal relationship between low shear rates akin to Newtonian fluids and high shear rates resembling power law fluids. Originating in 1972 [5], this model continues to hold widespread utility, particularly within chemical engineering, especially for viscoelastic materials. Termed as generalized Newtonian fluids, the Carreau fluid model provides an intricate yet comprehensive description, adept at predicting the behavior of polymer suspensions across diverse fluidic scenarios. With its versatility, the Carreau model finds itself at the heart of various applications, spanning from pulps and animal blood to molten polymers and toothpaste, among other similar materials. Its adaptability makes it a cornerstone in understanding and managing the flow behaviors of these substances. Over the years, numerous researchers have delved deep into the nuances of Carreau fluids, exploring their behavior across various morphologies and shedding light on their intricate characteristics. Hayat et al. [6] conducted a study to analyze the flow characteristics of Carreau liquid over a stretching sheet under convective conditions. Raju and Sandeep [7] provided numerical solutions for the flow of Casson-Carreau fluids over a stretching surface. Khan and Azam [8] investigated the numerical aspects of unsteady stretched flow and heat transfer of Carreau liquid over a sheet. Hayat et al. [9] employed the Homotopy Analysis Method (HAM) to obtain series solutions for the stretched flow problem of Carreau liquid. Raju et al. [10] discussed the numerical peculiarities associated with Carreau nanofluid confined in a cone. Additionally, Khan et al. [11] explored the magnetohydrodynamic flow of Carreau fluid over a variable stretching sheet and presented numerical solutions using the Keller box method. Both models have been examined to address the rheological gaps identified by Boyd et al. [12], particularly in scenarios lacking time effects and where flow is constrained by oscillation. The corresponding boundary layer approximations were tackled using the Lattice Boltzmann method. Gireesha et al. [13] delved into the nonlinear effects present in three-dimensional flows, specifically focusing on the interplay between Casson-Carreau fluid dynamics and various reactive components. Naga et al. [14] meticulously analyzed numerical data pertaining to three-dimensional flow, considering Casson-Carreau fluid as the base fluid and exploring mixtures with nanoparticles. Additionally, Nagendra et al. [15] investigated magnetized flow through a porous cylinder, where the presence of Casson-Carreau fluid with partial slip effects was a notable consideration.

In recent years, nanofluids have captured the attention of scientists worldwide due to their myriad engineering and industrial applications. These applications span across machining and electronics, engine cooling, air conditioning and refrigeration, heat exchange devices, fuel cells, solar water heating, and hybrid power systems, among others. Nanofluids, when compared to conventional fluids, exhibit exceptional thermal properties such as Brownian motion and thermophoresis. As a result, they are highly effective in enhancing heat transfer rates. This capability has led to their recognition as a promising alternative energy source. By incorporating nanoparticles with lengths on the order of 100 nanometers, the thermal conductivity of low-performing heat transfer fluids can be significantly improved. The term “nanofluid” was first introduced by Choi [16]. Building upon this foundation, numerous researchers have delved into the exploration of nanofluids across a wide range of geometries. Despite the promising characteristics of nanofluids, the issue of nanoparticle aggregation persists as a significant challenge in their practical application. In one work [17], Anwar used fractional relaxation times in the Buongiorno model to investigate transport processes in nanofluids. This study offers insight on the intricate relationship between nanoparticle dynamics and fluid flow properties. Anwar also investigated fractional nonlinear viscoelastic flow issues in his dissertation study [18], which contributed to a better understanding of nanofluid behavior under various flow circumstances. Collaborating with colleagues, Anwar delved into MHD nanofluid flow through porous media in the presence of thermal radiation and heat sources [19], [22]. These studies provide insights into the coupled effects of magnetic fields, thermal radiation, and heat sources on nanofluid behavior within porous structures, which are relevant to a range of engineering applications. In parallel, researchers such as Liping Yu et al. [20] investigated thermal optimization through the bioconvective jet flow of nanofluids, highlighting the potential for enhancing heat transfer in biological systems. Additionally, Hussain et al. [21] examined the effects of chemical reactions and melting heat on nanoliquid flow over curved surfaces, further expanding the understanding of nanofluid behavior in complex geometries. Furthermore, collaborative efforts such as the study by Muhammad Irfan et al. [23] explored the thermal performance of Joule heating and Soret-Dufour effects on nonlinear mixed convection radiative flow of Carreau nanofluids.

MHD pertains to the mechanical behavior of liquids under the influence of magnetic fields. It describes the motion of conducting liquids, where the presence of a magnetic field induces the generation of electric currents, thereby altering the mechanical properties of the liquid. MHD coupled with heat transfer finds extensive applications across various domains, including MHD peristaltic compressors, blood flow dynamics, wound treatments, pumping systems, turbo machinery, optical modulators, heat exchangers, cooling systems for nuclear reactors, and numerous other fields. Pavlov [24] presented a precise solution to the momentum equation governing MHD fluid flow along a stretched sheet. Subsequently, numerous scholars have delved into various aspects of MHD flow, often incorporating non-Newtonian models. For instance, Ahmed et al. [25] provided a numerical solution for MHD flow of a nanofluid with hyperbolic tangent properties over a non-linear stretching surface. Oğlakkaya and Bozkaya [26] investigated the effects of MHD on forced convection flow within an infinite channel containing a rotating cylinder. Sadighi et al. [27] conducted an analysis of heat and mass transfer in MHD nanofluid flow over a porous stretching sheet under specified boundary conditions. Additionally, Vidya et al. [28] explored entropy generation in MHD Casson fluid flow within an inclined channel with permeable walls, utilizing the Hermite Wavelet method for analysis. Hussain et al. [29] studied the effect of MHD on a steady two-dimensional flow of a Casson fluid towards a penetrable stretching wedge in the presence of suction/injection. In another attempt, Hussain et al. [30] examined the effect of thermal radiation on the Casson model-based flow at a time-independent two-dimensional MHD. They noticed skin friction enhances but the Nusselt number declines as the radiation parameter upsurges. Abideen and Saif [31] deliberated the effect of thermal radiation and internal heat generation on the flow of a Casson nanofluid adjacent to a curved and flexible surface containing a dispersion of carbon nanotubes (CNTs). The effect of the magnetic field and slip on the 2-phase fluctuating flow of Casson dusty fluid between inclined parallel plates has been examined by Khan et al. [32]. Saleem et al. [33] researchers examined the importance of the Darcy-Forchheimer Law and magnetic field in analyzing the comparison of Williamson-Casson fluid flow on an exponentially stretching sheet. Gireesha et al. [34] investigated the MHD boundary layer heat and mass transfer characteristics of a chemically reacting Casson fluid over a permeable stretching surface, considering non-uniform heat source/sink effects.

Stefan blowing, also known as wall suction/injection, is a technique utilized in fluid dynamics where a fluid is directed to impinge against or flow along a solid surface. This can occur due to various factors, such as injecting a fluid or the presence of an external flow that induces movement in that direction. The interaction between the fluid and the surface can significantly influence parameters like velocity, temperature, and other flow characteristics at the boundary. Stefan blowing finds numerous industrial applications, including drying and purification processes where boundaries are penetrated. In the context of fluid dynamics, several studies have explored the effects of surface blowing on mass transfer, particularly in scenarios resembling the Stefan problem. This phenomenon involves introducing blowing at the fluid-solid interface, which plays a crucial role in understanding complex heat transfer mechanisms. For instance, Kumar et al. [35] delved into the impacts of Soret and Dufour effects on Oldroyd-B fluid flow with Stefan blowing, contributing valuable insights into intricate heat transfer phenomena. Jyothi et al. [36] explored the significance of Stefan blowing effect on the flow and heat transfer of Casson nanofluid over a moving thin needle. They analyzed the convective flow behavior considering the Stefan blowing effect, providing insights into its influence on the transport phenomena. Puneeth et al. [37] investigated magneto-convective flow of Casson nanofluid induced by Stefan blowing in the presence of bio-active mixers. Moreover, Saleem and Tufail [38] delved into the analysis of an aligned magnetic nano-Casson fluid flow along with bio-convection, incorporating Stefan's blowing effect. Furthermore, Konai et al. [39] investigated the influences of Stefan blowing on the unsteady flow of Casson nanofluid past a stretching surface.

Microorganisms play a pivotal role in inducing a phenomenon known as “bioconvection” within fluids. This process arises from the collective motion of myriad tiny organisms freely moving within a fluid medium. Bioconvection can be categorized into chemotaxis or oxytactic, gyrotactic, and negative gravitaxis, each reflecting distinct behaviors of the microorganisms. Gyrotactic microorganisms, for instance, are commonly found in aquatic environments like oceans, ponds, and reservoirs, where they exhibit directed movement. These microorganisms move in a certain direction within the flowing liquid, driven by a combination of gravitational and viscous torque forces. As denser microorganisms tend to ascend towards the fluid surface, they contribute to the densification of the outer fluid layer, thereby creating instability and promoting bioconvection. The significance of bioconvection spans various applications such as biofuels, ethanol production, and eco-friendly industrial processes. Furthermore, the utilization of buoyant forces, along with microbes and nanoparticles, has emerged as a strategy to enhance bioconvection dynamics, particularly in dispersing nanoparticles within the fluid medium. Despite substantial advancements in sectors such as pharmaceuticals, environmental purification, microbe fuel production, microfluidic systems, and microbial nano-sensors, bioconvection remains largely unexplored. The ability of gyrotactic bacteria to move directionally within moving liquids adds complexity to this phenomenon, offering avenues for further investigation and potential applications across diverse fields. Garg et al. [40] provide a stability investigation of thermo-bioconvection flow of Jeffrey fluid with gravitactic microorganisms in an anisotropic porous medium. Their findings illustrate that an unstable system is established with an increase in the bioconvection Peclet number and microorganism concentration. According to Khaliq et al. [41], as the Peclet number and bioconvection Lewis number increase, the distribution of mobile microorganisms decreases. Akolade et al. [42] discussed the bioconvection phenomena in Eyring-Powell fluid, exhibiting composite characteristics of variable viscosity and motile microorganism density. Kaswan et al. [43] explored the bioconvection flow of magnetocross nanofluid with gyrotactic microorganisms and activation energy through an artificial neural network approach. Khan et al. [44] examined the optimization of entropy in bioconvection flow of Reiner-Rivlin nanoliquid with motile microorganisms. According to the many microbial species and their migration patterns, bioconvection technology can be categorized; for further information, read Mandal et al. [45]. Gyrotactic microorganism phenomena have recently been added to the Maxwell nanofluid flow by Manimekalai et al. [46]. Muhammad et al. [47] investigated the interplay between nanoparticles and mobile gyrotactic microorganisms in a MHD flow with Darcy-Forchheimer effects. Hussain et al. [48] examine the swimming behavior of gyrotactic microorganisms in the Williamson blood nanofluid model and a solar mimetic system over the peristaltic arterial wall. Adhikari and Das [49] explored the magnetized characteristics of Casson-Maxwell nanofluid coupled with microbes. Their study investigated the passage of these microbes within the Casson-Maxwell nanofluid past a tilted stretchy cylinder, with a focus on entropy optimization. In another study by Sarkar and Das [50], the swimming behavior of microorganisms was considered in conjunction with magneto-Sutterby nanofluid. The flow configuration involved a sliding cylinder within a Darcy-Forchheimer porous medium, incorporating Arrhenius energy effects. Expanding upon their previous work, Sarkar and Das [51] further augmented their model by incorporating nonlinear thermal radiation and stratification effects into the system.

Various methods are employed to tackle the complexity inherent in solving differential equations (DEs), which can be categorized as linear or nonlinear. Among these methods, one of the most prominent is proposed by Lie, which focuses on preserving the invariance of DEs possessing a continuous group of symmetries through Lie scaling transformations. This approach finds applications across a wide range of fields including topology, classical physics, relativity, geometry, and several other mathematical disciplines. In this context, Rosmil et al. [52] have dedicated their efforts to developing a group theoretic mathematical model describing the natural convection of nanofluids, which incorporates MHD, a stretched surface, porous medium, and thermal stratification. Similarly, Hamad et al. [53] have investigated a model involving radiation effects in stagnant flow over a flat surface, employing a Lie group theoretic approach with thermal convective boundary conditions. Furthermore, the exploration of rheological effects of the Prandtl-Erying fluid model over surfaces with heat and mass transfer utilizes a novel scaling group method [54]. Ferdows et al.'s study [55] employs a Lie group approach to investigate boundary layer flow of nanofluids over a horizontal flat plate within a Darcy porous medium, shedding light on the intricate dynamics of nanofluid behavior. Nabwey and El-Mkyn [56] apply Lie group analysis to understand thermophoresis on vertical surfaces in porous media, exploring particle motion and heat transfer phenomena. Additionally, Nabwey et al. [57] provide a comprehensive analysis of mixed convection stagnation-point flow of non-Newtonian nanofluids over a vertical stretching surface, which is critical in nanofluid research. Their work extends to the examination of unsteady slip flow over non-isothermal stretching sheets immersed in porous media, considering radiation and chemical reactions, as demonstrated by Nabwey, EL-Kabeir, and Rashad [58]. Furthermore, Bakier et al. [59] delve into magnetohydrodynamic mixed convection flow with melting effects, while EL-Kabeir, EL-Hakiem, and Rashad [60] investigate heat and mass transfer in MHD non-Darcy non-Newtonian natural convection adjacent to horizontal cylinders within saturated porous media.

The novelty and significance of our study lie in addressing a crucial gap in the literature concerning the unique combination of various complex phenomena. Specifically, we focus on the interplay of CCNF, chemically reactive species, over a stretching porous surface, while considering mass and heat transport rates alongside several additional factors. These factors include Stefan blowing, non-linear thermal radiation, heat source-sink effects, chemical reactions, thermophoretic and Brownian motions, convective conditions, Joule heating, motile microorganisms, and bioconvection, all within the framework of Lie group theory. By employing Lie group theory, we simplify mathematical models, analyze their symmetries, and generate numerical solutions [61], providing insights into the behavior of the system. To solve the intricate non-linear ODEs, we utilize the bvp4c technique [62], a proficient MATLAB built-in function, ensuring a thorough understanding of the system dynamics. Furthermore, we present tables and graphs to illustrate the behavior of dimensionless values, demonstrating that our predicted values align with prior findings in the literature under specific conditions. The developed flow model has practical applications across diverse industries, including chemical reactors, food pasteurization, wastewater treatment, and power plants. These applications offer opportunities for process optimization and enhanced performance in fluid flow and heat transfer processes.

The research is structured to address the following distinct aspects:

1. Develop a mathematical model incorporating various physical phenomena, including Stefan blowing, non-linear thermal radiation, heat source-sink, chemical reaction, thermophoretic and Brownian motions, convective conditions, Joule heating, and bioconvection.

2. Analyze the influence of microorganisms on controlling the suspension of nanomaterials within the nanofluid and evaluate their impact on flow behavior.

3. Investigate the effects of hydromagnetic forces on the momentum expression and comprehend their influence on flow characteristics.

4. Explore the potential enhancement of thermal efficiency in Casson- Carreau fluids by dispersing nanosized solid particles, thereby creating nanofluids.

5. Develop a set of nonlinear ODEs through Lie group scaling method and simplify the mathematical model for analysis.

6. Obtain reliable numerical solutions for the simplified ODEs using appropriate numerical techniques, such as the bvp4c scheme built-in function in MATLAB.

7. Validate the numerical results by comparing them with available computational data, ensuring the accuracy and reliability of the model predictions.

8. Investigate the influence of various flow parameters, including the mixed convection parameter, bioconvection Rayleigh and buoyancy ratio parameters, bioconvection Lewis number, and Peclet number, on flow characteristics, providing insights into the system's sensitivity to different factors.

## 2 Mathematical modeling

**Figure 1:**
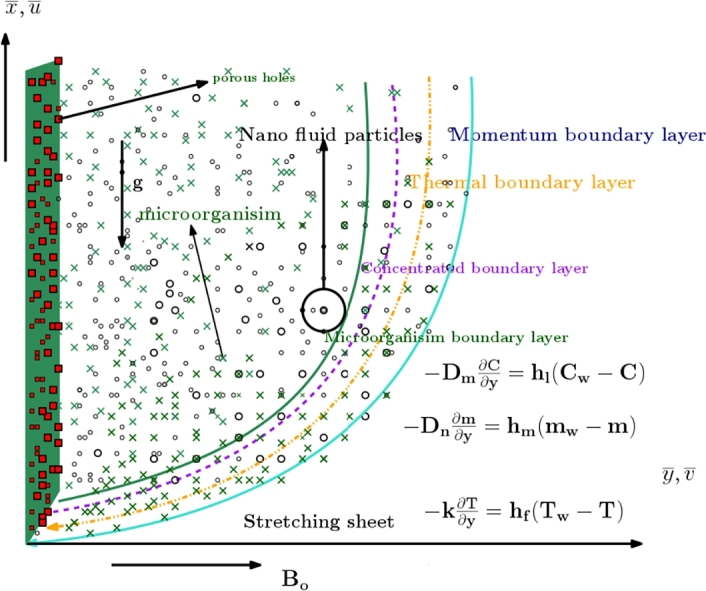
The flow geometry for Casson-Carreau nanofluid with Stefan blowing effects.

This problem involves investigating the flow of a non-Newtonian fluid over a porous vertical stretched flat surface. To accurately describe the fluid's behavior, we are utilizing the Casson-Carreau fluid models, which serves as the base fluid filled with nanoparticles. The fluid is assumed to be incompressible, with the flow restricted to the region where y>0. The stretching velocity, $u_{w}(x)$, is defined as *ax*, where *a* is a constant. Nonlinear thermal radiation, Joule heating and a heat source-sink's effects on energy transfer are incorporated into the analysis. The surface is maintained at a constant temperature $T_{w}$, while $T_{\infty }$ represents the ambient fluid temperature. Additionally, the gravitational acceleration *g* acts downward, and a perpendicular magnetic field $B_{0}$ is applied to the fluid motion (as illustrated in Fig. 1). The study introduces convective heating conditions for momentum, thermal, concentration, and motile aspects, enhancing the model's complexity. Furthermore, mixed bioconvection effects are considered, and the Boussinesq approximation is assumed, indicating that density variation primarily impacts the buoyancy term within the momentum equation. This addition contributes to the novelty of our analysis and allows us to examine the fluid flow in more detail.

The current analysis encompasses a comprehensive set of assumptions, including:

1. Utilization of the Casson and Carreau fluid model integrated with nanoparticles in steady frame.

2. Incorporation of porous medium effects, acknowledging the influence of permeability on flow behavior.

3. Implementation of Stefan blowing.

4. Evaluation of suction-injection effects, crucial for assessing fluid transport phenomena.

5. Addressing gravitational effects on a vertically stretched surface, recognizing their impact on flow patterns.

6. Examination of bioconvection phenomena induced by motile movement, adding biological relevance to the analysis.

7. Integration of nonlinear thermal radiation effects, considering complex heat transfer mechanisms.

8. Incorporation of heat source-sink effects, accounting for localized thermal energy generation or dissipation.

9. Consideration of Joule heating, acknowledging electrical energy conversion into heat within the system.

10. Evaluation of thermophoretic and Brownian motion, capturing particle transport mechanisms.

11. Assessment of chemical reaction dynamics, highlighting chemical transformations within the flow.

12. Application of boundary layer approximation techniques, facilitating the analysis of near-wall fluid behavior.

13. Consideration of convective heating effects, emphasizing the role of bulk fluid motion in heat transfer processes.

The stress tensor associated with the Casson-Carreau fluid model plays a crucial role in characterizing the fluid's behavior and its interaction with the porous stretched flat surface. By considering these various factors and equations, we aim to gain a comprehensive understanding of the complex phenomena occurring in this system. In this regard, the constitutive equation is [7]

(1) $$\overset{ˆ}{T}=-p\overset{ˆ}{I}+\tau_{C_{1}}+\tau_{C_{2}},$$

where *p* identifies as the pressure, $\overset{ˆ}{I}$ identifies as the identity tensor, $\tau_{C_{1}}$ identifies as the Cauchy stress tensor for the Casson fluid model and $\tau_{C_{2}}$ identifies as the Cauchy stress tensor for the Carreau fluid model. The shear stress tensor of the Casson fluid model incorporated as [1], [2], [3]:

(2) $$\tau_{C_{1}}=\left\{ \begin{array}{c} 2\left(\mu_{B}+\frac{p_{y}}{\sqrt{2\pi }}\right)e_{ij},\;\;\;\;\;\pi >\pi_{c}\\ 2\left(\mu_{B}+\frac{p_{y}}{\sqrt{2\pi }}\right)e_{ij},\;\;\;\;\;\pi <\pi_{c} \end{array}\right.,$$

where $\tau_{C_{1}}$ is elaborated as: $\pi =e_{ij}e_{ij}$, ($e_{ij}$ is the (i,j)th component of the deformation rate), *π* (product term)$,\;\pi_{c}$ (the critical value)$,\;\mu_{B}\;($the plastic dynamic viscosity) and $p_{y}$ (the yield stress) respectively. The shear stress tensor of the Carreau fluid model incorporated as [8], [9]

(3) $$\tau_{C_{2}}=\mu \left(\gamma_{1}\right){\overset{ˆ}{A}}_{1},$$

here, $\mu \left(\gamma_{1}\right)$ is defined by

(4) $$\mu \left(\gamma_{1}\right)=\mu_{\infty }+\left(\mu_{0}+\mu_{\infty }\right){\left[1+{\left(\Gamma \gamma_{1}\right)}^{2}\right]}^{\frac{n-1}{2}},$$

$\left(\mu_{0},\;\mu_{\infty }\right)$ zero-shear rate and the infinity shear-rate viscosities, respectively, Γ the material time constant, *n* the power law exponent, $\left(\frac{\mu -\mu_{\infty }}{\mu_{0}-\mu_{\infty }}\right)$ defines the slope in the power law region, ${\overset{ˆ}{A}}_{1}=\nabla \overset{ˆ}{V}+{\left(\nabla \overset{ˆ}{V}\right)}^{t}$ the first Rivlin-Erickson tensor, *t* represent the transpose and the shear rate is given by

(5) $$\gamma_{1}=\sqrt{\frac{1}{2}tr\left(A_{1}^{2}\right)}.$$

For considering the most practical cases, $\mu_{0}\gg \mu_{\infty }$, and $\mu_{\infty }=0$ taken to be zero. Consequently, in view of Eq. (5), Eq. (4) reduces to the following expression. One can obtain

(6) $$\mu \left(\gamma_{1}\right)=\mu_{0}{\left[1+{\left(\Gamma \gamma_{1}\right)}^{2}\right]}^{\frac{n-1}{2}}{\overset{ˆ}{A}}_{1}.$$

After using Eqs. ((1)-(3)), we obtain the following boundary layer equations:

(7) $$\frac{\partial u}{\partial x}+\frac{\partial v}{\partial y}=0,$$

(8) $$u\frac{\partial u}{\partial x}+v\frac{\partial u}{\partial y}=\upsilon \left(1+\frac{1}{\beta }\right)\frac{\partial^{2}u}{\partial y^{2}}+\upsilon \frac{3(n-1)}{2}\Gamma^{2}{\left(\frac{\partial u}{\partial y}\right)}^{2}\frac{\partial^{2}u}{\partial y^{2}}-\frac{\sigma B_{0}^{2}}{\rho }u-\frac{\upsilon }{k^{⁎}}\left(1+\frac{1}{\beta }\right)u+g\left[\begin{array}{c} \left(1-C_{\infty }\right)\beta_{T}(T-T_{\infty })-\left(\frac{\rho_{p}-\rho }{\rho }\right)(C-C_{\infty })\\ -\left(\frac{\rho_{m}-\rho }{\rho }\right)\beta_{m}(m-m_{\infty }) \end{array}\right],$$

$$u\frac{\partial T}{\partial x}+v\frac{\partial T}{\partial y}=\alpha_{m}\frac{\partial^{2}T}{\partial y^{2}}+\frac{Q_{o}}{{\left(\rho c_{p}\right)}_{f}}(T-T_{\infty })-\frac{1}{{\left(\rho c_{p}\right)}_{f}}\frac{\partial q_{r}}{\partial y}+D_{TC}\frac{\partial^{2}C}{\partial y^{2}}$$

(9) $$+\tau_{1}\left[D_{B}\frac{\partial C}{\partial y}\frac{\partial T}{\partial y}+\frac{D_{T}}{T_{\infty }}{\left(\frac{\partial T}{\partial y}\right)}^{2}\right]+\frac{\sigma B_{0}^{2}u^{2}}{{\left(\rho c_{p}\right)}_{f}}.$$

Where u,v are the velocity components in the *x* and *y*−directions, respectively. $\beta =\frac{\mu_{B}\sqrt{2\pi }}{p_{y}}$ represents the Casson fluid parameter, $B_{0}$ is the magnetic induction and $k^{⁎}$ signifies the permeability. The expression for non-linear thermal radiation, $q_{r}$, in the context of the Buongiorno model, is given by $q_{r}=-\frac{4\sigma^{⁎}}{3k^{⁎}}\frac{\partial T^{4}}{\partial y}=-\frac{16\sigma^{⁎}}{3k^{⁎}}T^{3}\frac{\partial T}{\partial y}$, where $\sigma^{⁎}$ represents the Stefan-Boltzmann constant and $k^{⁎}$ denotes the effective thermal conductivity. The temperature *T* is determined by $T=T_{\infty }\left\{1+\left(\theta_{w}-1\right)\theta \right\}$, where $T_{\infty }$ signifies the ambient temperature and $\theta_{w}$ represents the temperature ratio defined as $\frac{T_{w}}{T_{\infty }}$. This formulation captures the influence of temperature gradients on thermal radiation within the nanofluid, accounting for its complex flow characteristics and thermal behavior. In this context, $\alpha_{m}=\frac{k}{\rho c_{p}}$ denotes the thermal diffusivity, where *k* is the thermal conductivity, *ρ* represents density, and $c_{p}$ stands for specific heat capacity. The parameter $\tau_{1}=\frac{{\left(\rho c_{p}\right)}_{p}}{{\left(\rho c_{p}\right)}_{f}}$ is associated with the Buongiorno model, which characterizes the flow dynamics of the nanofluid. Here, ${\left(c_{p}\right)}_{f}$ represents the specific heat capacity for the fluid phase. The third term on the right hand side of Eq. (9) is as follows:

(10) $$\frac{\partial q_{r}}{\partial y}=\frac{\partial }{\partial y}\left(-\frac{16\sigma^{⁎}}{3k^{⁎}}{\left[T_{\infty }\left\{1+\left(\theta_{w}-1\right)\theta \right\}\right]}^{3}\frac{\partial \theta (T_{w}-T_{\infty })}{\partial y}\right),$$

(11) $$\frac{\partial q_{r}}{\partial y}=-\frac{16\sigma^{⁎}(T_{w}-T_{\infty })T_{\infty }^{3}}{3k^{⁎}}\left(3{\left\{1+\left(\theta_{w}-1\right)\theta \right\}}^{2}\left(\theta_{w}-1\right)\frac{\partial \theta }{\partial y}\frac{\partial \theta }{\partial y}+{\left[\left\{1+\left(\theta_{w}-1\right)\theta \right\}\right]}^{3}\frac{\partial^{2}\theta }{\partial y^{2}}\right).$$

This representation encapsulates the thermal transport properties and flow behavior essential for understanding the heat transfer mechanisms in nanofluid systems. The remaining boundary layer equations are:

(12) $$u\frac{\partial C}{\partial x}+v\frac{\partial C}{\partial y}=D_{m}\frac{\partial^{2}C}{\partial y^{2}}+\frac{D_{CT}}{T_{\infty }}\frac{\partial^{2}T}{\partial y^{2}}-kr(C-C_{\infty }),$$

(13) $$u\frac{\partial m}{\partial x}+v\frac{\partial m}{\partial y}=D_{n}\frac{\partial^{2}m}{\partial y^{2}}-\frac{bW_{c}}{C_{w}-C_{\infty }}\left[m\frac{\partial^{2}C}{\partial y^{2}}+\frac{\partial C}{\partial y}\frac{\partial m}{\partial y}\right].$$

We can identify the appropriate boundary conditions (BCs) [3−6] associated with Eqs. (7−13) as follows:

$$u=u_{w}(x),\;v=-\frac{D_{m}}{1-C_{w}}\frac{\partial C}{\partial y},\;-k\frac{\partial T}{\partial y}=h_{f}(T_{w}-T),\;-D_{m}\frac{\partial C}{\partial y}=h_{l}(C_{w}-C),$$

$$-D_{n}\frac{\partial m}{\partial y}=h_{m}(m_{w}-m),\;\;\text{at}\;\;\;y=0,$$

(14) $$u\to 0,\;T\to T_{\infty },\;C\to C_{\infty ,}\;m\to m_{\infty }\;\;\;\text{as}\;\;y\to \infty ,$$

with $u_{w}(x)=ax$, with a>0 representing the stretching surface, $v_{w}(x)=\frac{-D_{B}}{1-C_{w}}\frac{\partial C}{\partial y}\;\left(\text{Stefan blowing effects}\right)$ and $v_{w}(x)=-v_{w}$ (without Stefan blowing). In the given set of governing equations, we have defined several terms as follows: These include diffusion coefficients such as $D_{m},\;D_{n},\;D_{CT},\;D_{TC}$, which respectively represent Brownian diffusion, solutal diffusivity of the porous medium, diffusivity of oxygen by microorganisms in the fluid, and diffusivities of Soret and Dufour types. The influence of an applied uniform magnetic field $B_{0}$ is accounted for, while thermal expansion effects are quantified through thermal volumetric expansion coefficients $\beta_{T},\;\beta_{m}$. Fluid properties such as kinematic viscosity *υ*, density *ρ*, electrical conductivity *σ*, and specific heat $c_{p}$ are also considered, along with parameters related to heat generation-absorption $Q_{0}$ and thermal conductivity *k*. Velocity components u,v along the coordinate system, temperature *T*, temperature at the wall $T_{w}$, and free-stream temperature $T_{\infty }$ are essential variables in the analysis, as are concentration *C*, swimming cell speed $W_{c}$, and the concentration of microorganisms at the wall $m_{w}$ and in the free stream $m_{\infty }$. Furthermore, the fluid model employed in the analysis, known as the Carreau fluid, exhibits distinctive rheological behavior characterized by the parameter *n*. For 0<n<1, the fluid demonstrates shear-thinning or pseudoplastic behavior, meaning its viscosity decreases with increasing shear rate. Conversely, for n>1, the fluid displays shear-thickening or dilatant behavior, where viscosity increases with shear rate.

## 3 Dimensional reduction

In our analysis, we define several essential parameters and coefficients to capture the behavior of the system under study. One crucial concept we employ is dimensionality reduction, which involves transforming high-dimensional data into a more manageable and meaningful representation with fewer dimensions. This reduction is significant across various domains because it helps address challenges such as the curse of dimensionality and other drawbacks associated with high-dimensional spaces. By reducing dimensionality, we facilitate tasks such as classification, visualization, and data compression, making it easier to interpret and analyze complex data sets effectively. To make the system of equations (7)-(14) unitless, we consider the following:

(15) $$\overset{¯}{u}=\frac{u}{\sqrt{a\upsilon }},\;\overset{¯}{x}=\sqrt{\frac{a}{\upsilon }}x,\;\overset{¯}{v}=\frac{\nu }{\sqrt{a\upsilon }},\;\overset{¯}{y}=\sqrt{\frac{a}{\upsilon }}y,\;\chi =\frac{C-C_{\infty }}{C_{w}-C_{\infty }},\;\theta =\frac{T-T_{\infty }}{T_{w}-T_{\infty }},\;\Xi =\frac{m-m_{\infty }}{m_{w}-m_{\infty }}.$$

After applying the Eq. (15), we reached at the following:

(16) $$\frac{\partial \overset{¯}{u}}{\partial \overset{¯}{x}}+\frac{\partial \overset{¯}{v}}{\partial \overset{¯}{y}}=0.$$

(17) $$\overset{¯}{u}\frac{\partial \overset{¯}{u}}{\partial \overset{¯}{x}}+\overset{¯}{v}\frac{\partial \overset{¯}{u}}{\partial \overset{¯}{y}}=\left(1+\frac{1}{\beta }\right)\frac{\partial^{2}\overset{¯}{u}}{\partial {\overset{¯}{y}}^{2}}+\frac{3(n-1)}{2}\Gamma^{2}{\left(\frac{\partial \overset{¯}{u}}{\partial \overset{¯}{y}}\right)}^{2}\frac{\partial^{2}\overset{¯}{u}}{\partial {\overset{¯}{y}}^{2}}a^{2}-\sigma \frac{B_{0}^{2}}{\rho a}\overset{¯}{u}-\frac{\upsilon }{k^{⁎}a}\left(1+\frac{1}{\beta }\right)\overset{¯}{u}+\frac{g}{a\upsilon }\sqrt{\frac{\upsilon }{a}}\left[\begin{array}{c} \left(1-C_{\infty }\right)\beta_{T}(T-T_{\infty })-\left(\frac{\rho_{p}-\rho }{\rho }\right)(C-C_{\infty })\\ -\left(\frac{\rho_{m}-\rho }{\rho }\right)\beta_{m}(m-m_{\infty }) \end{array}\right].$$

Now, the thermal equation as:

$$\overset{¯}{u}\frac{\partial \theta }{\partial \overset{¯}{x}}+\overset{¯}{v}\frac{\partial \theta }{\partial \overset{¯}{y}}=\frac{k}{{\left(\rho c_{p}\right)}_{f}\upsilon }\frac{\partial^{2}\theta }{\partial {\overset{¯}{y}}^{2}}+\frac{Q_{0}}{{\left(\rho c_{p}\right)}_{f}a}\theta +\frac{D_{TC}}{\upsilon }\left(\frac{C_{w}-C_{\infty }}{T_{w}-T_{\infty }}\right)\frac{\partial^{2}\chi }{\partial {\overset{¯}{y}}^{2}}$$

$$+\frac{\tau_{1}D_{B}(C_{w}-C_{\infty })}{\upsilon }\frac{\partial \chi }{\partial \overset{¯}{y}}\frac{\partial \theta }{\partial \overset{¯}{y}}+\frac{\tau_{1}D_{T}(T_{w}-T_{\infty })}{T_{\infty }\upsilon }{\left(\frac{\partial \theta }{\partial \overset{¯}{y}}\right)}^{2}-\frac{\sigma B_{0}^{2}}{{\left(\rho c_{p}\right)}_{f}}\frac{{\overset{¯}{u}}^{2}\upsilon }{T_{w}-T_{\infty }}$$

(18) $$+\frac{1}{{\left(\rho c_{p}\right)}_{f}}\frac{16\sigma^{⁎}}{3k^{⁎}\upsilon }T_{\infty }^{3}\left(3{\left\{1+\left(\theta_{w}-1\right)\theta \right\}}^{2}\left(\theta_{w}-1\right){\left(\frac{\partial \theta }{\partial \overset{¯}{y}}\right)}^{2}+{\left[T_{\infty }\left\{1+\left(\theta_{w}-1\right)\theta \right\}\right]}^{3}\frac{\partial^{2}\theta }{\partial {\overset{¯}{y}}^{2}}\right).$$

(19) $$\overset{¯}{u}\frac{\partial \chi }{\partial \overset{¯}{x}}+\overset{¯}{v}\frac{\partial \chi }{\partial \overset{¯}{y}}=\frac{D_{m}}{\upsilon }\frac{\partial^{2}\chi }{\partial {\overset{¯}{y}}^{2}}+\frac{D_{CT}}{\upsilon T_{\infty }}\frac{T_{w}-T_{\infty }}{C_{w}-C_{\infty }}\frac{\partial^{2}\theta }{\partial {\overset{¯}{y}}^{2}}-\frac{kr}{a}\chi .$$

(20) $$\overset{¯}{u}\frac{\partial \Xi }{\partial \overset{¯}{x}}+\overset{¯}{v}\frac{\partial \Xi }{\partial \overset{¯}{y}}=\frac{D_{m}}{\upsilon }\frac{\partial^{2}\Xi }{\partial {\overset{¯}{y}}^{2}}-\frac{bw_{c}}{\upsilon }\left[\frac{\partial^{2}\chi }{\partial {\overset{¯}{y}}^{2}}\Xi +\frac{\partial \chi }{\partial \overset{¯}{y}}\frac{\partial \Xi }{\partial \overset{¯}{y}}\right].$$

The unitless BCs are:

(21) $$\overset{¯}{u}=\overset{¯}{x},\overset{¯}{v}=\frac{\left(C_{w}-C_{\infty }\right)}{1-C_{w}}\frac{D_{B}}{\upsilon }\frac{\partial \chi }{\partial \overset{¯}{y}},\;\frac{\partial \theta }{\partial \overset{¯}{y}}=\frac{h_{f}}{k}\sqrt{\frac{a}{\upsilon }}\left(\theta -1\right),\;\frac{\partial \chi }{\partial \overset{¯}{y}}=\frac{h_{l}}{D_{m}}\sqrt{\frac{a}{\upsilon }}(\chi -1),\frac{\partial \Xi }{\partial \overset{¯}{y}}=\frac{h_{m}}{D_{n}}\sqrt{\frac{a}{\upsilon }}(\Xi -1)\;\text{at }\overset{¯}{y}=0\;\overset{¯}{u}\to 0,\;\theta \to 0,\;\chi \to 0,\;\Xi \to 0\;\text{as }\overset{¯}{y}\to \infty .$$

After dropping the bar in Eqs. ((16)-(22)), we used the stream function as:

(22) $$u=\frac{\partial \psi }{\partial y},v=-\frac{\partial \psi }{\partial y},$$

$$\left(\frac{\partial \psi }{\partial y}\frac{\partial^{2}\psi }{\partial x\partial y}-\frac{\partial \psi }{\partial x}\frac{\partial^{2}\psi }{\partial y^{2}}\right)=\left(1+\frac{1}{\beta }\right)\frac{\partial^{3}\psi }{\partial y^{2}}+\frac{3(n-1)}{2}\Gamma^{2}{\left(\frac{\partial^{2}\psi }{\partial y^{2}}\right)}^{2}\frac{\partial^{3}\psi }{\partial y^{3}}a^{2}$$

(23) $$-\left(\frac{\sigma B_{0}^{2}}{\rho a}+\frac{\upsilon }{k^{⁎}a}\left(1+\frac{1}{\beta }\right)\right)\frac{\partial \psi }{\partial y}+\frac{g}{a\upsilon }\sqrt{\frac{a}{\upsilon }}\left(1-C_{\infty }\right)\beta_{T}(T_{w}-T_{\infty })\left[\begin{array}{c} \theta -\frac{\left(\rho_{p}-\rho \right)(C_{w}-C_{\infty })}{\rho \left(1-C_{\infty }\right)\beta_{T}(T_{w}-T_{\infty })}\chi \\ -\frac{\left(\rho_{m}-\rho \right)(m_{w}-m_{\infty })\beta_{m}}{\rho \left(1-C_{\infty }\right)\beta_{T}(T_{w}-T_{\infty })}\Xi \end{array}\right],$$

here, the dimensionless parameters are: $M=\frac{\sigma B_{0}^{2}}{\rho a}$, $D_{a}=\frac{\upsilon }{k^{⁎}a}$.

$$\left(\frac{\partial \psi }{\partial y}\frac{\partial \theta }{\partial x}-\frac{\partial \psi }{\partial x}\frac{\partial \theta }{\partial y}\right)=\frac{k}{\rho \upsilon c_{p}}\frac{\partial^{2}\theta }{\partial y^{2}}+\frac{Q_{0}}{\rho c_{p}a}\theta +\frac{D_{TC}}{\nu }\frac{C_{w}-C_{\infty }}{T_{w}-T_{\infty }}\frac{\partial^{2}\chi }{\partial y^{2}}+$$

$$+\frac{\tau_{1}D_{B}(C_{w}-C_{\infty })}{\upsilon }\frac{\partial \chi }{\partial \overset{¯}{y}}\frac{\partial \theta }{\partial \overset{¯}{y}}+\frac{\tau_{1}D_{T}(T_{w}-T_{\infty })}{T_{\infty }\upsilon }{\left(\frac{\partial \theta }{\partial \overset{¯}{y}}\right)}^{2}-\frac{\sigma B_{0}^{2}}{{\left(\rho c_{p}\right)}_{f}}\frac{\upsilon }{\left(T_{w}-T_{\infty }\right)}{\left(\frac{\partial \psi }{\partial y}\right)}^{2}$$

(24) $$+\frac{1}{{\left(\rho c_{p}\right)}_{f}}\frac{16\sigma^{⁎}}{3k^{⁎}\upsilon }T_{\infty }^{3}\left(3{\left\{1+\left(\theta_{w}-1\right)\theta \right\}}^{2}\left(\theta_{w}-1\right){\left(\frac{\partial \theta }{\partial y}\right)}^{2}+{\left[\left\{1+\left(\theta_{w}-1\right)\theta \right\}\right]}^{3}\frac{\partial^{2}\theta }{\partial y^{2}}\right).$$

Moreover, in the above equation the dimensionless parameters are: $\mathrm{Pr}=\frac{\upsilon {\left(\rho c_{p}\right)}_{f}}{k}=\frac{\upsilon }{\alpha_{m}},\;Q=\frac{Q_{0}}{a{\left(\rho c_{p}\right)}_{f}},\;N_{b}=\frac{\tau_{1}D_{B}(C_{w}-C_{\infty })}{\upsilon },\;Nt=\frac{\tau_{1}D_{T}(T_{w}-T_{\infty })}{T_{\infty }\upsilon },\;R=\frac{16\sigma^{⁎}}{3kk^{⁎}\upsilon }T_{\infty }^{3},\;E_{c}=\frac{a\upsilon }{c_{p}\left(T_{w}-T_{\infty }\right)},\;\Omega =\frac{g}{a\upsilon }\sqrt{\frac{a}{\upsilon }}\left(1-C_{\infty }\right)\beta_{T}(T_{w}-T_{\infty }),\;Nr=\frac{\left(\rho_{p}-\rho \right)(C_{w}-C_{\infty })}{\rho \left(1-C_{\infty }\right)\beta_{T}(T_{w}-T_{\infty })}$ and $Rb=\frac{\left(\rho_{m}-\rho \right)(m_{w}-m_{\infty })\beta_{m}}{\rho \left(1-C_{\infty }\right)\beta_{T}(T_{w}-T_{\infty })}$.

(25) $$\frac{\partial \psi }{\partial y}\frac{\partial \chi }{\partial x}-\frac{\partial \psi }{\partial x}\frac{\partial \chi }{\partial y}=\frac{D_{m}}{\upsilon }\frac{\partial^{2}\chi }{\partial y^{2}}+\frac{D_{CT}}{\upsilon T_{\infty }}\frac{T_{w}-T_{\infty }}{C_{w}-C_{\infty }}\frac{\partial^{2}\theta }{\partial y^{2}}-\frac{kr}{a}\chi ,$$

(26) $$\frac{\partial \psi }{\partial y}\frac{\partial \Xi }{\partial x}+\frac{\partial \psi }{\partial x}\frac{\partial \Xi }{\partial y}=\frac{D_{n}}{\upsilon }\frac{\partial^{2}\Xi }{\partial y^{2}}-\frac{bW_{c}}{\upsilon }\left[\frac{\partial^{2}\chi }{\partial y^{2}}\Xi +\frac{\partial \Xi }{\partial y}\frac{\partial \chi }{\partial y}\right],$$

with BCs

(27) $$\frac{\partial \psi }{\partial y}=x,\frac{\partial \psi }{\partial x}=\frac{\left(C_{w}-C_{\infty }\right)}{1-C_{w}}\frac{D_{B}}{\upsilon }\frac{\partial \chi }{\partial y},\;\frac{\partial \theta }{\partial y}=\frac{h_{f}}{k}\sqrt{\frac{a}{\upsilon }}\left(\theta -1\right),\frac{\partial \chi }{\partial y}=\frac{h_{l}}{D_{m}}\sqrt{\frac{a}{\upsilon }}(\chi -1),\;\frac{\partial \Xi }{\partial y}=\frac{h_{m}}{D_{n}}\sqrt{\frac{a}{\upsilon }}(\Xi -1)\;\text{at }y=0\;\frac{\partial \psi }{\partial y}\to 0,\;\theta \to 0,\;\chi \to 0,\;\Xi \to 0\;\text{as }y\to \infty .$$

## 4 One-parameter Lie scaling transformations

Lie's symmetry approach to DEs is a powerful method that involves identifying tactics for solving DEs by considering symmetry groups. This approach has found applications across a wide range of mathematical domains, including mechanics, physics, and various applied sciences. Numerous published results [53], [54], [55] in these fields highlight the efficacy of Lie's theory in addressing nonlinear problems represented as DEs. The central objective of Lie's symmetry analysis is to discover one or more parameters associated with local continuous transformations that preserve the equations' form. Subsequently, these parameters are manipulated to derive reductions, invariant solutions, or similarity solutions. This approach has proven its utility through extensive usage by researchers in diverse contexts. Another valuable application within this framework is the conditional symmetry technique. A crucial aspect of Lie's approach involves the study of infinitesimal transformations and the corresponding structure of the Lie algebra. This study is closely tied to the examination of multi-parameter Lie groups of transformations. It's worth noting that one-parameter Lie group transformations can be seen as the exponentiation of infinitesimal generators and are a subset of multi-parameter Lie group transformations. Each multi-parameter transformation corresponds to a distinct infinitesimal generator. Let *ε* be the small one-parameter of the scaling transformation [54], [55].

(28) $$Ϝ:x^{⁎}=xe^{\varepsilon r_{1}},\;y^{⁎}=ye^{\varepsilon r_{2}},\;\psi^{⁎}=\psi e^{\varepsilon r_{3}},\;\theta^{⁎}=\theta e^{\varepsilon r_{4}},\;\chi^{⁎}=\chi e^{\varepsilon r_{5}},\;\Gamma^{⁎}=\Gamma e^{\varepsilon r_{6}},\;\Xi^{⁎}=\Xi e^{\varepsilon r_{7}}\;h_{f}^{⁎}=h_{f}e^{\varepsilon r_{8}},\;h_{l}^{⁎}=h_{l}e^{\varepsilon r_{9}},\;h_{m}^{⁎}=h_{m}e^{\varepsilon r_{10}},\;\Omega^{⁎}=\Omega e^{\varepsilon r_{11}},$$

where $r_{1,}\;r_{2,}\;r_{3}...r_{11}$ are arbitrary real numbers. The coordinates $\left(x,\;y,\;\psi ,\;\theta ,\;\chi ,\;\Gamma ,\;\Xi ,\;h_{f},\;h_{l},\;h_{m},\;\Omega \right)$ were changed into $\left(x^{⁎},\;y^{⁎},\;\psi^{⁎},\;\theta^{⁎},\;\chi^{⁎},\;\Gamma^{⁎},\;\Xi^{⁎},\;h_{f}^{⁎},\;h_{l}^{⁎},\;h_{m}^{⁎},\;\Omega^{⁎}\right)$ using the point transformation shown in (28). Consequently, applying Eq. (28) to Eqs. (22−27) yields the following:

$$e^{\varepsilon (r_{1}+2r_{2}-2r_{3})}\left(\frac{\partial \psi^{⁎}}{\partial y^{⁎}}\frac{\partial^{2}\psi^{⁎}}{\partial x^{⁎}\partial y^{⁎}}-\frac{\partial \psi^{⁎}}{\partial x^{⁎}}\frac{\partial^{2}\psi^{⁎}}{\partial y^{{⁎}^{2}}}\right)=e^{\varepsilon (3r_{2}-r_{3})}\left(1+\frac{1}{\beta }\right)\frac{\partial^{3}\psi^{⁎}}{\partial y^{{⁎}^{3}}}$$

(29) $$+\frac{3(n-1)}{2}{\left(\Gamma^{⁎}\right)}^{2}a^{2}{\left(\frac{\partial^{2}\psi^{⁎}}{\partial y^{{⁎}^{2}}}\right)}^{2}\frac{\partial^{3}\psi^{⁎}}{\partial y^{{⁎}^{3}}}e^{\varepsilon (7r_{2}-3r_{3}-2r_{6})}$$

(30) $$-\left(M^{2}+D_{a}\left(1+\frac{1}{\beta }\right)\right)e^{\varepsilon (r_{2}-r_{3)}}\frac{\partial \psi^{⁎}}{\partial y^{⁎}}+\Omega^{⁎}\left[\begin{array}{c} \theta^{⁎}e^{-\varepsilon \left(r_{4}+r_{11}\right)}-Nr\chi^{⁎}e^{-\varepsilon \left(r_{5}+r_{11}\right)}\\ -Rb\Xi^{⁎}e^{-\varepsilon \left(r_{7}+r_{11}\right)} \end{array}\right].$$

$$e^{\varepsilon (r_{1}+r_{2}-r_{3}-r_{4})}\left(\frac{\partial \psi^{⁎}}{\partial y^{⁎}}\frac{\partial \theta^{⁎}}{\partial x^{⁎}}-\frac{\partial \psi^{⁎}}{\partial x^{⁎}}\frac{\partial \theta^{⁎}}{\partial y^{⁎}}\right)=\frac{1}{\mathrm{Pr}}e^{\varepsilon (2r_{2}-r_{4})}\frac{\partial^{2}\theta^{⁎}}{\partial y^{{⁎}^{2}}}$$

$$+\frac{Q_{0}}{{\left(\rho c_{p}\right)}_{f}a}e^{\varepsilon (-r_{4})}\theta^{⁎}+e^{\varepsilon \left(2r_{2}-r_{5}\right)}\frac{D_{TC}}{\nu T_{\infty }}\left(\frac{C_{w}-C_{\infty }}{T_{w}-T_{\infty }}\right)\frac{\partial^{2}\chi^{⁎}}{\partial y^{{⁎}^{2}}}+Nbe^{\varepsilon \left(2r_{2}-r_{4}-r_{5}\right)}\frac{\partial \chi^{⁎}}{\partial y^{⁎}}\frac{\partial \theta^{⁎}}{\partial y^{⁎}}$$

$$+Nt{\left(\frac{\partial \theta^{⁎}}{\partial y^{⁎}}\right)}^{2}e^{\varepsilon \left(2r_{2}-r_{4}\right)}-M^{2}E_{c}{\left(\frac{\partial \psi^{⁎}}{\partial y^{⁎}}\right)}^{2}e^{\varepsilon \left(2r_{3}-2r_{2}\right)}+$$

(31) $$+\frac{1}{{\left(\rho c_{p}\right)}_{f}}\frac{16\sigma^{⁎}}{3k^{⁎}}T_{\infty }^{3}\left[\begin{array}{c} 3\left\{\begin{array}{c} e^{\varepsilon \left(-2r_{4}+2r_{2}\right)}+{\left(\theta_{w}-1\right)}^{2}{\left(\theta^{⁎}\right)}^{2}e^{\varepsilon \left(2r_{2}-4r_{4}\right)}\\ +2\left(\theta_{w}-1\right)\theta^{⁎}e^{\varepsilon \left(2r_{2}-3r_{4}\right)} \end{array}\right\}\left(\theta_{w}-1\right){\left(\frac{\partial \theta^{⁎}}{\partial y^{⁎}}\right)}^{2}+\\ \left\{\begin{array}{c} e^{\varepsilon \left(-r_{4}+2r_{2}\right)}+{\left(\theta_{w}-1\right)}^{3}{\left(\theta^{⁎}\right)}^{3}e^{\varepsilon \left(-4r_{4}+2r_{2}\right)}\\ +3{\left(\theta_{w}-1\right)}^{2}{\left(\theta^{⁎}\right)}^{2}e^{\varepsilon \left(-3r_{4}+2r_{2}\right)}+3\left(\theta_{w}-1\right)\left(\theta^{⁎}\right)e^{\varepsilon \left(-2r_{4}+2r_{2}\right)} \end{array}\right\}\frac{\partial^{2}\theta^{⁎}}{\partial y{⁎}^{2}} \end{array}\right],$$

$$e^{\varepsilon \left(r_{1}+r_{2}-r_{3}-r_{4}\right)}\left(\frac{\partial \psi^{⁎}}{\partial y⁎}\frac{\partial \chi^{⁎}}{\partial x^{⁎}}-\frac{\partial \psi^{⁎}}{\partial x^{⁎}}\frac{\partial \chi^{⁎}}{\partial y^{⁎}}\right)=e^{\varepsilon \left(2r_{2}-r_{5}\right)}\frac{D_{m}}{\upsilon }\frac{\partial^{2}\chi^{⁎}}{\partial y^{{⁎}^{2}}}+e^{\varepsilon \left(2r_{2}-r_{4}\right)}\frac{D_{CT}}{\upsilon T_{\infty }}\left(\frac{T_{w}-T_{\infty }}{C_{w}-C_{\infty }}\right)\frac{\partial^{2}\chi^{⁎}}{\partial y^{{⁎}^{2}}}$$

(32) $$-\frac{kr}{a}\chi^{⁎}e^{\varepsilon \left(-r_{5}\right)}$$

(33) $$e^{\varepsilon (r_{1}+r_{2-}r_{3-}r_{7})}\left(\frac{\partial \psi^{⁎}}{\partial y^{⁎}}\frac{\partial \Xi^{⁎}}{\partial x^{⁎}}-\frac{\partial \psi^{⁎}}{\partial x^{⁎}}\frac{\partial \Xi^{⁎}}{\partial y^{⁎}}\right)=\frac{D_{n}}{\upsilon }\frac{\partial^{2}\Xi^{⁎}}{\partial y^{⁎}}e^{\varepsilon (2r_{2}-r_{7})}-\frac{bW_{c}}{\upsilon }\left[\begin{array}{c} \frac{\partial^{2}\chi^{⁎}}{\partial y^{{⁎}^{2}}}\Xi^{⁎}e^{\varepsilon (2r_{2}-r_{5}-r_{7})}\\ +\frac{\partial \chi^{⁎}}{\partial y^{⁎}}\frac{\partial \Xi^{⁎}}{\partial y^{⁎}}e^{\varepsilon (2r_{2}-r_{5}-r_{7})} \end{array}\right]$$

and the BCs are:

(34) $$\frac{\partial \psi^{⁎}}{\partial y^{⁎}}e^{\varepsilon (r_{2}-r_{3})}=e^{\varepsilon (-r_{1})}x^{⁎},\;\frac{\partial \psi^{⁎}}{\partial x^{⁎}}e^{\varepsilon (r_{1}-r_{3})}=e^{\varepsilon (r_{2}-r_{5})}\frac{\left(C_{w}-C_{\infty }\right)}{1-C_{w}}\frac{D_{B}}{\upsilon }\frac{\partial \chi^{⁎}}{\partial y^{⁎}},\;\frac{\partial \theta^{⁎}}{\partial y^{⁎}}e^{\varepsilon (r_{2}-r_{4})}=\frac{-h_{f}^{⁎}}{k}\sqrt{\frac{a}{\upsilon }}\left(e^{\varepsilon (-r_{8})}-\theta^{⁎}e^{\varepsilon (-r_{4}-r_{8})}\right),\;\frac{\partial \chi^{⁎}}{\partial y^{⁎}}e^{\varepsilon (r_{2}-r_{5})}=\frac{-h_{l}^{⁎}}{D_{m}}\sqrt{\frac{a}{\upsilon }}(e^{\varepsilon (-r_{9})}-\chi^{⁎}e^{\varepsilon (-r_{6}-r_{9})}),\frac{\partial \Xi^{⁎}}{\partial y^{⁎}}e^{\varepsilon (r_{2}-r_{7})}=\frac{-h_{m}^{⁎}}{D_{n}}\sqrt{\frac{a}{\upsilon }}(e^{\varepsilon (-r_{10})}-\Xi^{⁎}e^{\varepsilon (-r_{7}-r_{10})})\;\text{at }y^{⁎}=0\;\frac{\partial \psi^{⁎}}{\partial y^{⁎}}\to 0,\;\theta^{⁎}\to 0,\;\chi^{⁎}\to 0,\;\Xi^{⁎}\to 0\;\text{as }y^{⁎}\to \infty .$$

Now, the condition where the system of Eqs. (29)-(33) will remain invariant under *Ϝ* is that the equations that link the exponents *r*'s are

(35) $$r_{1}+2r_{2}-2r_{3}=3r_{2}-r_{3}=7r_{2}-3r_{3}-2r_{6}=r_{2}-r_{3}=-r_{4}-r_{11}=-r_{5}-r_{11}=-r_{7}-r_{11}.$$

(36) $$r_{1}-r_{2}-r_{3}-r_{4}=2r_{2}-r_{4}=-r_{4}=2r_{2}-r_{5}=2r_{2}-r_{4}-r_{5}=2r_{2}-r_{4}=2r_{2}-r_{5}=2r_{2}-2r_{4}=2r_{2}-4r_{4}=2r_{2}-3r_{4}.$$

(37) $$r_{1}+r_{2}-r_{3}-r_{5}=2r_{2}-r_{5}=2r_{2}-r_{4}=-r_{5}.$$

(38) $$r_{1}+r_{2}-r_{3}-r_{7}=2r_{2}-r_{7}=2r_{2}-r_{5}-r_{7}=2r_{2}-r_{5}.$$

From the BCs (34), we get the following

(39) $$-r_{3}+r_{1}=-r_{5}+r_{2},\;-r_{3}+r_{2}=-r_{1},\;-r_{4}+r_{2}=-r_{8}=-r_{8}-r_{4},-r_{5}+r_{2}=-r_{9}=-r_{9}+r_{5},\;-r_{7}+r_{2}=-r_{10}+r_{7}$$

From Eq. (35), we get

(40) $$r_{1}=r_{3},\;r_{2}=0,$$

Eqs. (36)-(38) yields

(41) $$r_{4}=r_{5}=r_{7}=r_{8}=r_{9}=r_{10}=0.$$

by using above Eqs. (40)-(41), we get easily the following relation

(42) $$r_{6}=-r_{1},\;r_{11}=r_{1}.$$

By using the values of *r*'s (Eqs. (40)-(42)) in Eq. (28), we get:

(43) $$F:x^{⁎}=xe^{\varepsilon r_{1}},\;y^{⁎}=y,\;\psi^{⁎}=\psi e^{\varepsilon r_{1}},\;\theta^{⁎}=\theta ,\;\chi^{⁎}=\chi ,\;\Gamma^{⁎}=\Gamma e^{-\varepsilon r_{1}},\;\Xi^{⁎}=\Xi ,h_{f}^{⁎}=h_{f},\;h_{l}^{⁎}=h_{l},\;h_{m}^{⁎}=h_{m},\;\Omega^{⁎}=\Omega e^{\varepsilon r_{1}}.$$

By utilizing Taylor's series expansion on transformation given in Eq. (43), we get the characteristics equation as:

(44) $$\frac{dx^{⁎}}{x^{⁎}r_{1}}=\frac{dy^{⁎}}{0}=\frac{d\psi^{⁎}}{\psi^{⁎}r_{1}}=\frac{d\theta^{⁎}}{0}=\frac{d\chi^{⁎}}{0}=\frac{d\Gamma^{⁎}}{-\Gamma^{⁎}r_{1}}=\frac{d\Xi^{⁎}}{0}=\frac{dh_{f}^{⁎}}{0}=\frac{dh_{l}^{⁎}}{0}=\frac{dh_{m}^{⁎}}{0}=\frac{d\Omega^{⁎}}{\Omega^{⁎}r_{1}}.$$

One can easily obtain, from Eq. (44), the new similarity transformation as given in the following:

(45) $$y^{⁎}=\xi ,\;\chi^{⁎}=\chi (\xi ),\;\theta^{⁎}=\theta (\xi ),\;\psi^{⁎}=x^{⁎}f(\xi ),\;\Gamma^{⁎}={\left(x^{⁎}\right)}^{-1}\Gamma_{0,}\;\Xi^{⁎}=\Xi (\xi ),\;h_{f}^{⁎}=h_{f}(\xi )\;h_{l}^{⁎}=h_{l}(\xi ),\;\;h_{m}^{⁎}=h_{m}(\xi ),\;\;\Omega^{⁎}=x^{⁎}\Omega .$$

By using Eqs. (40)-(42), (45) into the system of equations (29)-(34), finally we reached

(46) $$\left(1+\frac{1}{\beta }\right)f^{{}^{\prime \prime \prime }}-{\left(f^{{}^{\prime }}\right)}^{2}+f^{{}^{\prime \prime }}f+\frac{3\left(n-1\right)}{2}We{\left(f^{{}^{\prime \prime }}\right)}^{2}f^{{}^{\prime \prime \prime }}-\left(M^{2}+D_{a}\left(1+\frac{1}{\beta }\right)\right)f^{\overset{´}{}}+\Omega (\theta -Nr\chi -Rb\Xi )=0.$$

with $\beta =We=a^{2}\Gamma^{2}$ the Weissenberg number, $M^{2}=\frac{\sigma B^{2}}{\rho a}$ is the Hartmann number, *n* is the power law index, $D_{a}=\frac{v}{R^{⁎}a}$ stands for Darcy number $\Omega =\frac{g\left(1-C_{\infty }\right)\beta_{T}(T_{w}-T_{\infty })}{\upsilon \sqrt{a\upsilon }}$ is the mixed bioconvection parameter$,\;Nr=\frac{\left(\rho_{p}-\rho \right)(C_{w}-C_{\infty })}{\rho \left(1-C_{\infty }\right)\beta_{T}(T_{w}-T_{\infty })}$ is the bioconvection Rayleigh number, and $Rb=\frac{\left(\rho_{m}-\rho \right)(m_{w}-m_{\infty })\beta_{m}}{\rho \left(1-C_{\infty }\right)\beta_{T}(T_{w}-T_{\infty })}$ denote the buoyancy ratio parameter respectively. By definition

(47) $$R_{e}=\frac{u_{w}x}{\upsilon }=\frac{\left(ax\right)x}{\upsilon },$$

after using the Eq. (15) in Eq. (47), we have

(48) $$R_{e}={\left(\overset{¯}{x}\right)}^{2},$$

after dropping bar in Eq. (48)

(49) $$\sqrt{R_{e}}=x.$$

By the use of Eq. (49)

$$\theta^{{}^{\prime \prime }}+Q\theta \mathrm{Pr}+Nd\chi^{{}^{\prime \prime }}+\mathrm{Pr}[N_{b}\chi^{{}^{\prime }}\theta^{{}^{\prime }})+Nt{\left(\theta^{{}^{\prime }}\right)}^{2}]+R[{\left\{1+\left(\theta_{w}-1\right)\theta \right\}}^{3}\theta^{{}^{\prime \prime }}$$

(50) $$+3(\theta_{w}-1){\left(\theta^{{}^{\prime }}\right)}^{2}{\left(1+(\theta_{w}-1)\theta \right)}^{2}]+\mathrm{Pr}f\theta^{{}^{\prime }}-M^{2}E_{c}R_{e}\mathrm{Pr}{\left(f^{\prime }\right)}^{2}=0,$$

where $\mathrm{Pr}=\frac{\upsilon }{\alpha_{m}}$ is the Prandtl number $Nd=\frac{D_{TC}}{\alpha_{m}}\frac{C_{w}-C\infty }{T_{W-}T_{\infty }}$ is modified Dufour parameter, $Q=\frac{Q_{0}}{\rho c_{p}a}$ is a source-sink parameter, $N_{b}=\frac{\tau_{1}D_{B}(C_{w}-C_{\infty })}{\nu }$ the Brownian parameter, and $Nt=\frac{\tau_{1}D_{t}(T_{w}-T_{\infty })}{T_{\infty }\nu }$ the thermophoresis parameter.

(51) $$\chi^{{}^{\prime \prime }}+S_{c}f\chi^{{}^{\prime }}+Ld\theta^{{}^{\prime \prime }}-S_{c}K_{r}\chi =0,$$

with $S_{c}=\frac{\nu }{D_{b}}$ the Schmidt number, $Ld=\frac{D_{CT}}{\alpha_{m}T_{\infty }}\frac{T_{W-}T_{\infty }}{C_{w}-C\infty }$ the modified Soret number and $K_{r}=\frac{kr}{a}$ is the chemical reaction parameter.

(52) $$\Xi^{{}^{\prime \prime }}+L_{b}\left(f\Xi^{{}^{\prime }}-Pe\left[\chi^{{}^{\prime \prime }}\Xi +\chi^{{}^{\prime }}\Xi^{{}^{\prime }}\right]\right)=0,$$

with $Pe=\frac{LW_{c}}{D_{m}}$, and $L_{b}=\frac{\nu }{D_{n}}$. The BCs are:

(53) $$f^{{}^{\prime }}=1,\;f=\frac{S}{S_{c}}\chi^{{}^{\prime }}\left(0\right),\;\theta^{{}^{\prime }}\left(0\right)=B_{i1}(\theta \left(0\right)-1),\;\chi^{{}^{\prime }}\left(0\right)=B_{i2}\left(\chi \left(0\right)-1\right),\;\Xi^{{}^{\prime }}\left(0\right)=B_{i3}(\Xi (0)-1),f^{{}^{\prime }}\to \infty ,\theta \to 0,\chi \to 0,\Xi \to 0\;\text{as }\xi \to \infty ,$$

with $S=\frac{\left(C_{w}-C_{\infty }\right)}{1-C_{w}},\;B_{i1}=\frac{h_{f}^{⁎}}{k}\sqrt{\frac{a}{\upsilon }},\;B_{i2}=\frac{h_{l}^{⁎}}{D_{m}}\sqrt{\frac{a}{\upsilon }},\;B_{i3}=\frac{h_{m}^{⁎}}{D_{n}}\sqrt{\frac{a}{\upsilon }}$. The parameters are named as Stefan blowing, Biot number for temperature, concentration and motile organism.

## 5 Physical quantities

It is crucial to comprehend specific physical data in fluid mechanics. The explanation of several elements of fluid dynamics, including mass transport, flow patterns, heat transfer, and the density of moving creatures, depends heavily on these numbers. Within the energy systems area, we have incorporated the new uses of our studied problem into heat-related apparatus, greatly increasing the efficiency of nuclear reactors, heating and cooling systems, and radiators. In the pharmaceutical sector, particularly in the manufacturing of generic medications, we have taken advantage of their versatility and heat tolerance. These uses include developing biosensor technology and enhancing boiler gas outlet efficiency. In addition, the following categories have been established for the local expressions of skin friction coefficient (Cf) in the case of CCNF model, local Nusselt number $\left(Nu_{x}\right)$, local Sherwood number $\left(Sh_{x}\right)$, and for the motile microorganisms density number $\left(Nn_{x}\right)$.

(54) $$C_{f}=\frac{\tau_{w}}{\rho {\left(a\overset{¯}{x}\right)}^{2}},$$

(55) $$Sh_{x}=\frac{\overset{¯}{x}p_{m}}{D_{m}(C_{w}-C_{\infty })},$$

(56) $$Nu_{x}=\frac{\overset{¯}{x}q_{w}}{k(T_{w}-T_{\infty })},$$

(57) $$Nn_{x}=\frac{\overset{¯}{x}g_{s}}{D_{n}(n_{w}-n_{\infty })},$$

with

(58) $$p_{m}=-D_{m}{\left(\frac{\partial C}{\partial \overset{¯}{y}}\right)}_{\overset{¯}{y}=0},$$

(59) $$q_{w}=-k{\left(\frac{\partial T}{\partial \overset{¯}{y}}\right)}_{\overset{¯}{y}=0}+q_{r},$$

(60) $$g_{s}=-D_{n}{\left(\frac{\partial n}{\partial \overset{¯}{y}}\right)}_{\overset{¯}{y}=0}.$$

The shear stress for Carreau fluid [8], [9]

(61) $$\tau_{w}={\left(\frac{\partial \overset{¯}{u}}{\partial \overset{¯}{y}}\right)}_{\overset{¯}{y}=0}+\frac{(n-1){\left(We\right)}^{2}}{2}{\left(\frac{\partial \overset{¯}{u}}{\partial \overset{¯}{y}}\right)}_{\overset{¯}{y}=0}^{3}.$$

The shear stress for Casson fluid [1], [2], [3]

(62) $$\tau_{w}=\mu_{\beta }\left(1+\frac{1}{\beta }\right){\left(\frac{\partial \overset{¯}{u}}{\partial \overset{¯}{y}}\right)}_{\overset{¯}{y}=0}.$$

By using Eq. (15) into Eqs (54)-(62), we reached at

(63) $$\frac{Nu_{x}}{\sqrt{R_{e}}}=-\left[1+R{\left\{1+\left(\theta_{w}-1\right)\theta \right\}}^{3}\right]\theta^{\prime }\left(0\right),=-B_{i1}(\theta \left(0\right)-1)\left[1+R{\left\{1+\left(\theta_{w}-1\right)\theta \left(0\right)\right\}}^{3}\right]$$

(64) $$\frac{Sh_{x}}{\sqrt{R_{e}}}=-\chi^{\prime }\left(0\right),=-B_{i2}\left(\chi \left(0\right)-1\right)$$

(65) $$\frac{Nn_{x}}{\sqrt{R_{e}}}=-\Xi^{\prime }\left(0\right).=-B_{i3}(\Xi (0)-1)$$

The skin friction for Carreau and Casson fluid are:

(66) $$\sqrt{R_{e}}C_{f}=f^{\prime \prime }\left(0\right)+\frac{(n-1)We}{2}{\left(f^{\prime \prime }\left(0\right)\right)}^{3},$$

(67) $$\sqrt{R_{e}}C_{f}=f^{\prime \prime }\left(0\right)\left(1+\frac{1}{\beta }\right).$$

## 6 Method of solution and numerical results

**Figure 2:**
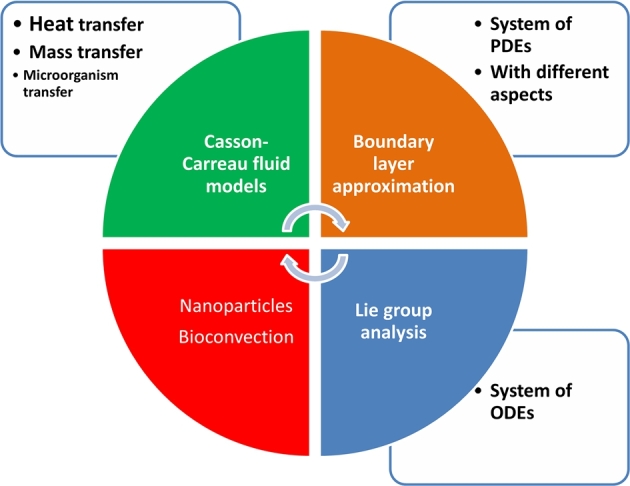
Computational model for Non-Newtonian magneto Casson-Carreau Nanofluid (CCNF).

The results or findings section is supported by the numerical technique applied to the system of ODEs produced by the Lie scaling method. Following the derivation of these findings, we proceed to the discussion section. The bvp4c technique [61] is used to achieve the numerical solution of Eqs. (46), (50)-(52) supported by the restricting constraints Eq. (53). Through graphical and tabular data for the velocity field, temperature distribution, concentration of nanoparticles, and rescaled density of motile microorganisms, the effects of effective parameters that are involved in the current scrutiny are displayed. Current flow issues have dimensionless ODEs that have a highly nonlinear character. The primary research issue is to precisely solve problems involving non-linear systems of flows. To obtain the numerical solution of the current model, the built-in numerical method known as bvp4c under the commercial program MATLAB is more appropriate [62]. The Lobbato-IIIa formula is implemented using a separate code called the bvp4c scheme. Initially the procedure, higher order differential equations for velocity, temperature, volumetric concentration, and motile microbes are each reduced to the first-order flow issue. In particular, Fig. 2 provides a visual exploration of the outlined assumptions within various flow transport scenarios, as depicted in Fig. 1. To convert the given equations for the bvp4c code, we rewrite them as a system of first-order ODEs:

(68) $$f=g_{1},\;f^{\prime }=g_{2},\;f^{\prime \prime }=g_{3},\;f^{\prime \prime \prime }=g_{3}^{\prime },\theta =g_{4},\;\theta^{\prime }=g_{5},\;\theta^{\prime \prime }=g_{5}^{\prime },\chi =g_{6},\;\chi^{\prime }=g_{7},\;\chi^{\prime \prime }=g_{7}^{\prime },\Xi =g_{8},\;\Xi^{\prime }=g_{9},\;\Xi^{\prime \prime }=g_{9}^{\prime },$$

By using the Eq. (68) into the Eqs. (46), (50)-(53)

(69) $$g_{3}^{\prime }=\left[\frac{-1}{(1+\frac{1}{\beta })+\frac{3}{2}\left(n-1\right)Weq_{3}^{2}}\right]\left[g_{3}g_{1}-{\left(g_{2}\right)}^{2}-(M^{2}+D_{a}(1+\frac{1}{\beta }))g_{2}+\cos\alpha \Omega (g_{4}-Nrg_{6}-Rbg_{8})\right],$$

(70) $$g_{5}^{\prime }=-\left[\begin{array}{c} Q\mathrm{Pr}g_{4}+R[{\left(1+(\theta_{w}-1)g_{4}\right)}^{3}g_{5}^{\prime }+3(\theta_{w}-1)g_{5}^{2}{\left(1+(\theta_{w}-1)g_{4}\right)}^{2}]+g_{7}^{\prime }\mathrm{Pr}Nd+\\ \mathrm{Pr}N_{b}g_{7}g_{5}+\mathrm{Pr}Ntg_{5}^{2}+\mathrm{Pr}g_{1}g_{5}-M^{2}E_{c}\mathrm{Pr}R_{e}{\left(g_{2}\right)}^{2} \end{array}\right],$$

(71) $$q_{7}^{\prime }=-\left[S_{c}g_{1}g_{7}+\frac{N_{t}}{N_{b}}g_{5}^{\prime }-g_{6}K_{r}S_{c}\right],$$

(72) $$q_{9}^{\prime }=-\left[L_{b}g_{9}g_{1}-Pe[(g_{8})g_{7}^{\prime }+g_{7}g_{9}]\right],$$

with BCs

(73) $$\xi =0,\;g_{2}=1,\;g_{1}=\frac{S}{S_{c}}g_{7}\left(0\right),\;g_{5}=Bi_{1}\left(g_{4}\left(0\right)-1\right),\;g_{7}=Bi_{2}\left(g_{6}\left(0\right)-1\right),\;g_{9}=Bi_{1}\left(g_{8}\left(0\right)-1\right)\xi \to \infty ,\;g_{2}\to 0,\;g_{4}\to 0,\;g_{6}\to 0,\;g_{8}\to 0.$$

With the assistance of Eqs. (69)-(73), we derive both graphical results and numerical solutions presented in Table 1, Table 2, Table 3, Table 4, Table 5, Table 6, Table 7. Employing the values specified in Eqs. (63)-(67), we obtain corresponding values for the Table 1, Table 2, Table 3, Table 4, Table 5, Table 6, Table 7. Table 1 illustrates the numerical variations of the Ω,Nr,Rb concerning key physical quantities (local expressions of skin friction coefficient $\left(Cf_{x}\right)$ in the case of CCNF model within the flow configurations. The rates of change for *Nr* in both models are 0.0007 and 0.0005, while for Ω, they are -0.0754 and -0.0536, respectively. Similarly, the rate of change for *Rb* in both models is -0.002 and -0.0002. Analyzing these numerical results reveals that the impact of the Bioconvection Rayleigh number is positive in both models, with a notably higher rate observed for the Casson fluid compared to the Carreau fluid model. For the buoyancy ratio parameter, the rate of change remains consistent across both models. However, in the case of the mixed bioconvection parameter, the reduction in impact is more pronounced for the Casson fluid model compared to the Carreau fluid model. Table 2 illustrates the behavior of the Biot number concerning thermal, concentration, and motile organism aspects. For $B_{i1}$, a significant decrease is observed in the Nusselt number, while there is an evident increase in the Sherwood number and motile density number. In the case of $B_{i2}$, there is a noticeable increase in the Sherwood number, while the other two quantities exhibit a decreasing rate of change. Conversely, for $B_{i3}$, a notable rate of change is observed in the motile density number, showing rapid decay, while the other two quantities exhibit slower growth compared to the motile density number.

**Table 1:** Numerical values of the skin friction coefficient of CCNF models for the variation of Ω, *Nr*, *Rb* with *θ*_*w*_ = 1.5, *β* = 4, (In case of Casson *We* = 0) $M=D_{a}=0.5,\;n=3,\;S=0.5,\;S_{c}=4,\;\mathrm{Pr}=2=L_{b}=Pe=K_{r},\;Q=0=E_{c}=Nd$*N*_*b*_ = 0.3, *Nt* = 1 *Bi*_1_ = 0.1 = *Bi*_2_ = *Bi*_3_ otherwise (*We* = 0.4, *β* = ∞).

*Nr*	Ω	*Rb*	$-f^{\prime \prime }\left(0\right)\left(1+\frac{1}{\beta }\right)$	$-\left(f^{\prime \prime }\left(0\right)+\frac{(n-1)We}{2}{\left(f^{\prime \prime }\left(0\right)\right)}^{3}\right)$
**0.1**	0.1	0.1	2.725205614	2.236098239
**0.3**	0.1	0.1	2.725563200	2.236365307
**0.5**	0.1	0.1	2.725920731	2.236632335
0.1	**0.1**	0.1	2.725205614	2.236098239
0.1	**0.3**	0.1	2.686920066	2.208982180
0.1	**0.5**	0.1	2.649766330	2.182492921
0.1	0.1	**0.1**	2.725205614	2.236098239
0.1	0.1	**0.3**	2.725092522	2.236020350
0.1	0.1	**0.5**	2.724979428	2.235942464

**Table 2:** Numerical values of the Nusselt number, Sherwood number and motile density number for the variations of *B*_*i*1_, *B*_*i*2_, *B*_*i*3_.

*B*_*i*1_	*B*_*i*2_	*B*_*i*3_	$\frac{Nu_{x}}{\sqrt{R_{e}}}$	$\frac{Sh_{x}}{\sqrt{R_{e}}}$	$\frac{Nn_{x}}{\sqrt{R_{e}}}$
**0.1**	0.1	0.1	0.171394069	0.096585611	0.092397505
**0.3**	0.1	0.1	0.361737656	0.097178268	0.092480318
**0.5**	0.1	0.1	0.441766212	0.097643502	0.092512490
0.1	**0.1**	0.1	0.171394069	0.096585611	0.092397505
0.1	**0.3**	0.1	0.170222692	0.272776270	0.094720286
0.1	**0.5**	0.1	0.169128810	0.428830512	0.095855997
0.1	0.1	**0.1**	0.171394069	0.096585611	0.092397505
0.1	0.1	**0.3**	0.171395466	0.096585496	0.240609128
0.1	0.1	**0.5**	0.171396528	0.096585480	0.354261781

**Table 3:** Comparison of the skin friction $-f^{\prime \prime }\left(0\right)\left(1+\frac{1}{\beta }\right)$ for the variations of *M*, *β* and all other parameters are zero.

*β*	*M*	Hayat et. al. [1]	Gireesha et al. [34]	Present Values
**0.8**	0.5	1.67705	1.67712	1.677050983
**1.4**	0.5	1.46385	1.46386	1.463850109
**2.0**	0.5	1.36931	1.36931	1.369306394
**3.0**	0.5	1.29099	1.29099	1.290994449
0.8	**0**	1.22475	−	1.500000001
0.8	**0.6**	1.42829	−	1.749285568
0.8	**1.2**	1.91312	−	2.343074903
0.8	**1.7**	2.41557	−	2.958462438

**Table 4:** Numerical values of the skin friction coefficient $-f^{\prime \prime }\left(0\right)\left(1+\frac{1}{\beta }\right)$ for the variation of *M*, *β* without Stefan blowing effects and other parameters are zero.

*β*	*M*	*S*	Shahzad et al. [2]	Present values
**0.5**	0.5	0.5	2.20256	2.202562419
**0.8**	0.5	0.5	1.94558	1.945582496
**1.3**	0.5	0.5	1.75799	1.757991532
**2.0**	0.5	0.5	1.64195	1.641941091
0.8	**0**	0.5	1.77069	1.770690633
0.8	**0.6**	0.5	2.01706	2.017059705
0.8	**1.2**	0.5	2.60638	2.606374334
0.8	**1.5**	0.5	2.96570	2.965695123
0.8	0.5	**0**	1.67705	1.677050983
0.8	0.5	**0.7**	2.06318	2.063184170
0.8	0.5	**1.4**	2.51728	2.517278185
0.8	0.5	**2.0**	2.95256	2.952562419

**Table 5:** Numerical values of $-\chi^{\prime }\left(0\right)$ with the variations of the Sherwood number, chemical reaction and all other parameters are zero.

*Kr*	*Sc*	Shehzad et. al. [2]	Present values
0.01	1	0.59136	0.591355196
0.1	1	0.66898	0.668980545
1.0	1	1.17650	1.176499870
10	1	3.23175	3.231227959

**Table 6:** Numerical values of $-f^{\prime \prime }\left(0\right)$ with *n* = 1, *S* = 0 and all other parameters are zero.

*M*	Hayat et al. [9]	Present values
0	1.0000	1.000000
0.2	1.01980	1.019803903
0.5	1.11803	1.118033989
0.8	1.28063	1.280624847
1.0	1.41421	1.414213562
1.2	1.56205	1.562049935
1.5	1.80303	1.802775638

**Table 7:** Numerical values of $-\left(f^{\prime \prime }\left(0\right)+\frac{(n-1)We}{2}{\left(f^{\prime \prime }\left(0\right)\right)}^{3}\right)$ in case of the Carreau fluid model and *n* = 3 all other parameters are zero.

*M*	*We*	*S*	Hayat et al. [9]	Present values
**0.0**	0.2	0.5	1.297262158	1.59061
**0.4**	0.2	0.5	1.374979970	1.71786
**0.8**	0.2	0.5	1.583318492	2.08031
**0.2**	**0**	**0.5**	**1.300000000**	**1.30000**
0.2	**0.4**	0.5	1.427206733	1.70379
0.2	**0.6**	0.5	1.532344011	1.62281
0.2	0.2	**0**	1.041167240	1.18806
0.2	0.2	**0.4**	1.273383639	1.52363
0.2	0.2	**0.8**	1.548827647	1.96023

## 7 Evaluating current results against previous numerical findings

In this section, Table 3, Table 4, Table 5, Table 6, Table 7 present the validation of the numerical results against previous published papers. Table 3 elaborates on the comparison of the skin friction coefficient for Casson fluid, with the effects of Carreau fluid considered negligible. There is good agreement between the results of Hayat et al. [1] and Gireesha et al. [34] regarding the Casson parameter, even when considering the effects of MHD. However, accuracy is lacking for the variation of the MHD parameter compared to Hayat et al. [1], with no results available for Gireesha et al. [34]. These results do not account for the effects of porosity, such as suction-injection phenomena S=0. Table 4 demonstrates agreement with the results of Shahzad et al. [2], an extension of the work by Hayat et al. [1], particularly regarding suction effects. Accuracy is observed not only for the Casson parameter but also for MHD and suction-injection cases. Table 5 presents a comparison of the Sherwood number, considering negligible effects of CCNF. Results align accurately with those published in [2], especially regarding variations in chemical reaction and Schmidt number. In Table 6, good results are obtained for MHD, similar to those seen in Hayat et al. [9], when neglecting CCNF effects. However, Table 7 does not yield accurate results for the Carreau fluid model when considering negligible effects of the Casson fluid model. Accurate results are only obtained when choosing We=0. Based on these numerical findings, further comparisons with previous works [1], [9] are necessary.

## 8 Discussion of the results

The discussion section integrates the results and findings, leading to the conclusion and recommendations. It is grounded in the graphical representation of novel parameters. This section also addresses the limitations of the results across various ranges. Specifically, the discussion section is tailored to analyze velocity, momentum, thermal behavior, concentration, and microorganism dynamics. The graphical representations in this segment showcase the interplay of various results for different parameter values with $\mathrm{Pr}=2,\;\theta_{w}=1.5,\;n=3,\;We=0.4,\;S_{c}=1,\;M=0.5,\;\Omega =Rb=Nr=0.1,\;B_{i1}=B_{i2}=B_{i3}=0.1,\;Nd=0.1,\;N_{b}=0.3,\;Q=0.01,\;R=1,\;E_{c}=0.01,\;Lb=1,\;Pe=1,\;K_{r}=1,\;Nt=0.3,\;D_{a}=0.1,\;\beta =4$. These parameters are named as follows: Prandtl number, temperature ratio parameter, power-law index, Weissenberg number, Schmidt number, Hartmann number, mixed bioconvection parameter, bioconvection Rayleigh number, buoyancy ratio parameter, Biot number for the thermal, concentration and motile organism, Dufour parameter, Brownian motion parameter, heat generation-absorption parameter, thermal radiation parameter, Eckert number, Lewis number for motile organisms, Peclet number, chemical reaction, thermophoresis, Darcy number and Casson parameter.

### 8.1 Graphical discussion of velocity profile for novel parameters

**Figure 3:**
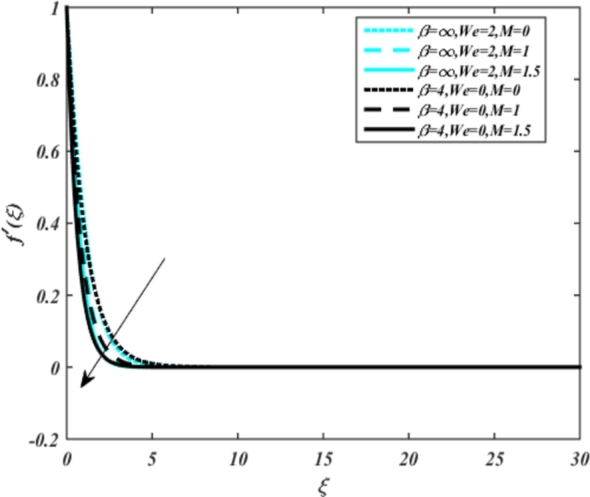
Occurrences of *f*′(*ξ*) for *M* embedded with CCNF.

**Figure 4:**
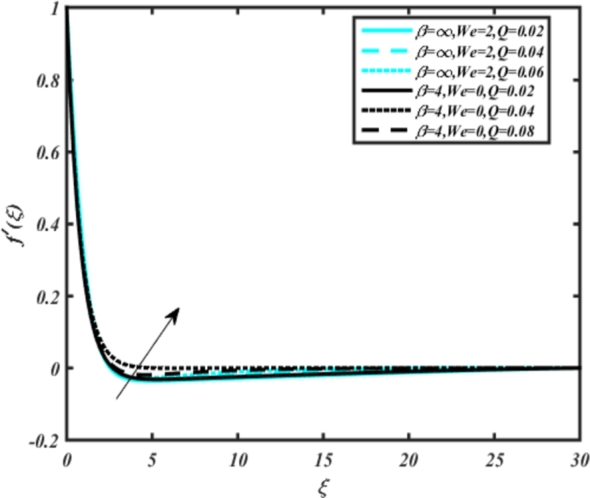
Occurrences of *f*(*ξ*) for *Q* embedded with CCNF.

**Figure 5:**
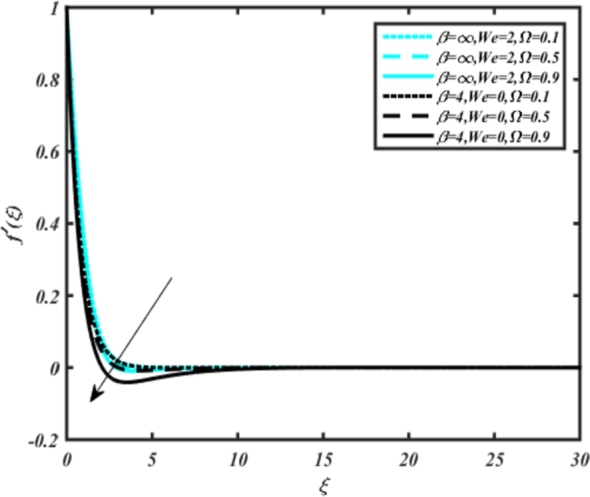
Occurrences of *f*(*ξ*) for Ω embedded with CCNF.

**Figure 6:**
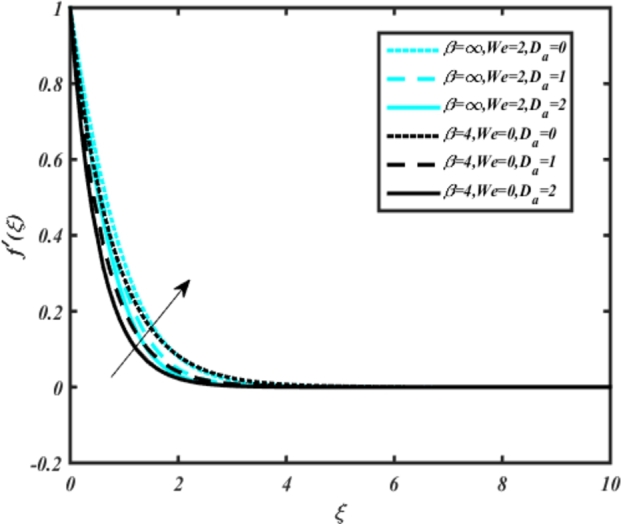
Occurrences of *f*(*ξ*) for *D*_*a*_ embedded with CCNF.

In all graphs, blue lines represent Carreau fluid effects, while black lines represent Casson fluid effects. In Fig. 3, the influence of the magnetic parameter *M* on velocity fields for both Carreau and Casson phases is depicted. Increasing *M* values lead to a depreciation of velocity profiles for both fluid models due to fluctuations in the Lorentz force induced by the magnetic field. This drag force increases resistance to transport processes, operating in the opposite direction to flow. Fig. 4 illustrates the impact of the heat source-sink parameter *Q* on velocity fields. It's evident that larger *Q* values result in increased velocity for both fluid models. Heat generation plays a pivotal role in heat transfer and thermodynamics, involving the internal production of thermal energy within a material or system from various physical processes. Figure 5, Figure 6 demonstrate the effects of the mixed bio-convection parameter Ω and the Darcy parameter $D_{a}$ on velocity distributions, respectively. Porous media contain void spaces through which fluid flows, with the porosity parameter representing the fraction of void space in the material's volume. According to Darcy's law, fluid velocity is inversely proportional to the permeability of the medium, with higher porosity typically leading to higher permeability and increased fluid flow velocity. The mixed bio-convection parameter decreases momentum velocity for both Casson and Carreau fluids, while the Darcy parameter enhances fluid velocity, as observed in Fig. 6.

### 8.2 Graphical discussion of temperature profile for novel parameters

**Figure 7:**
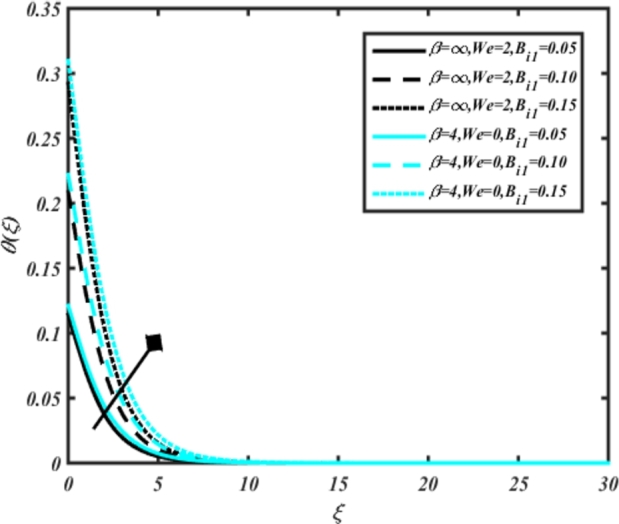
Occurrences of *θ*(*ξ*) for *B*_*i*1_ embedded with CCNF.

**Figure 8:**
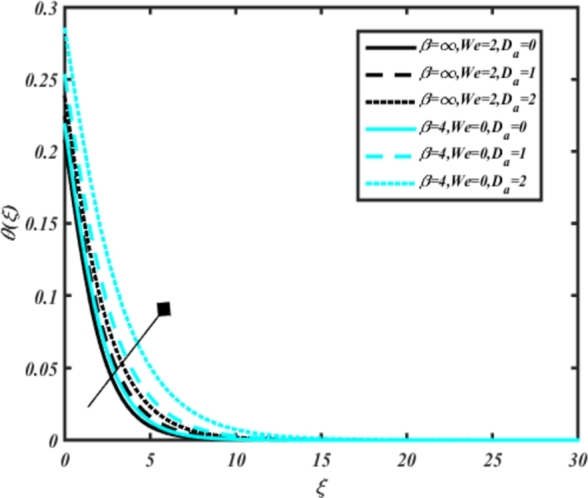
Occurrences of *θ*(*ξ*) for *D*_*a*_ embedded with CCNF.

**Figure 9:**
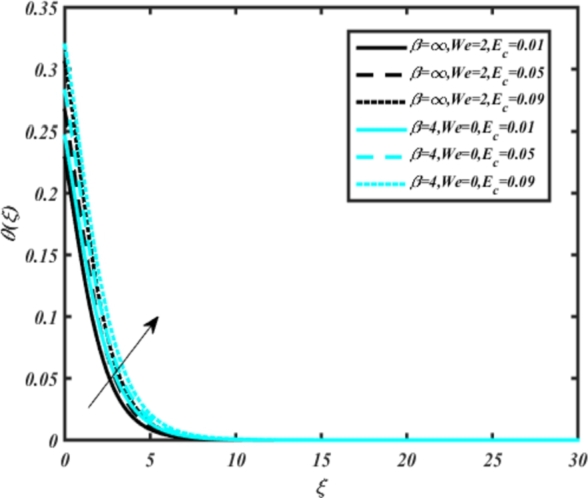
Occurrences of *θ*(*ξ*) for *E*_*c*_ embedded with CCNF.

**Figure 10:**
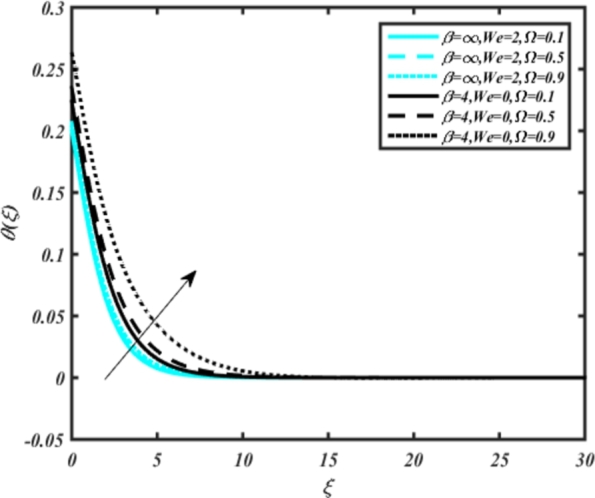
Occurrences of *θ*(*ξ*) for Ω embedded with CCNF.

**Figure 11:**
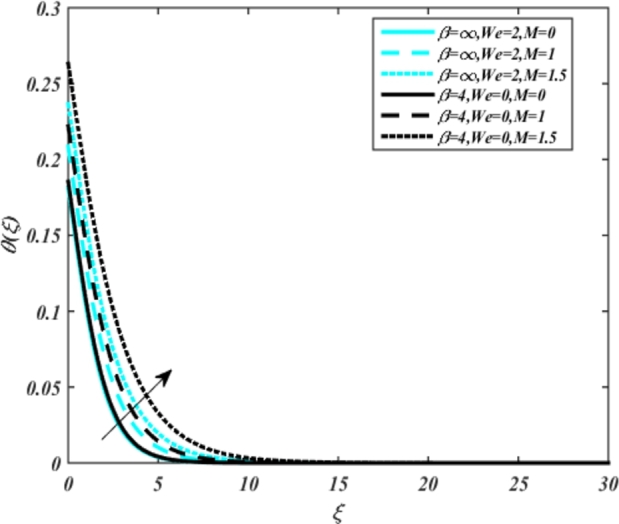
Occurrences of *θ*(*ξ*) for *M* embedded with CCNF.

**Figure 12:**
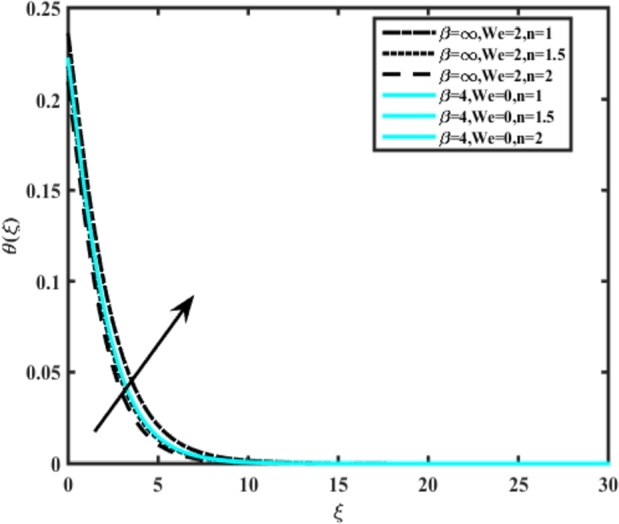
Occurrences of *θ*(*ξ*) for *n* embedded with CCNF.

**Figure 13:**
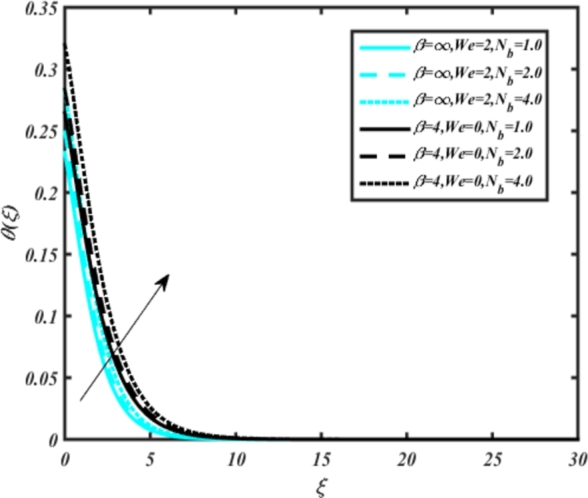
Occurrences of *θ*(*ξ*) for *N*_*b*_ embedded with CCF.

**Figure 14:**
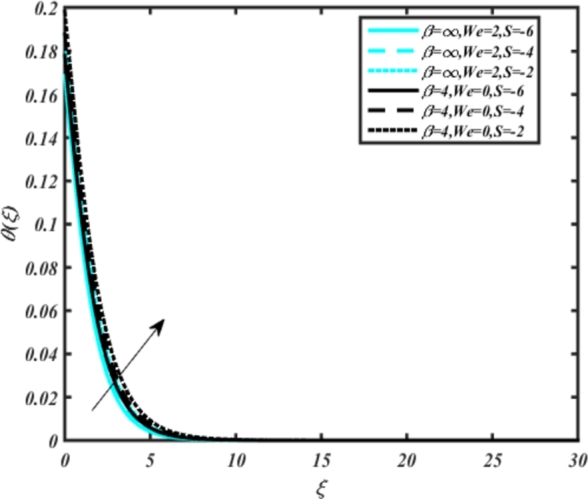
Occurrences of *θ*(*ξ*) for *S* embedded with CCF.

**Figure 15:**
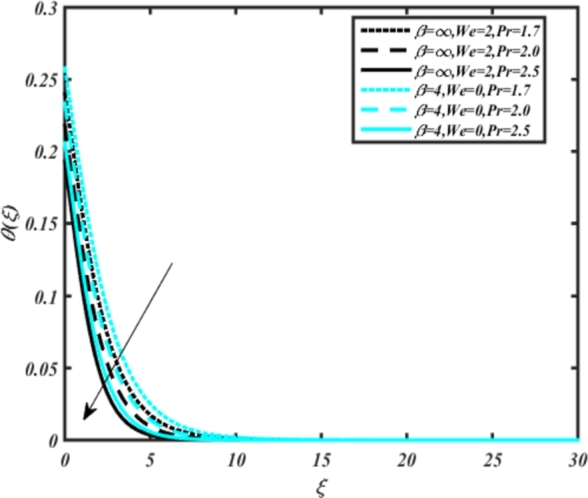
Occurrences of *θ*(*ξ*) for Pr embedded with CCNF.

**Figure 16:**
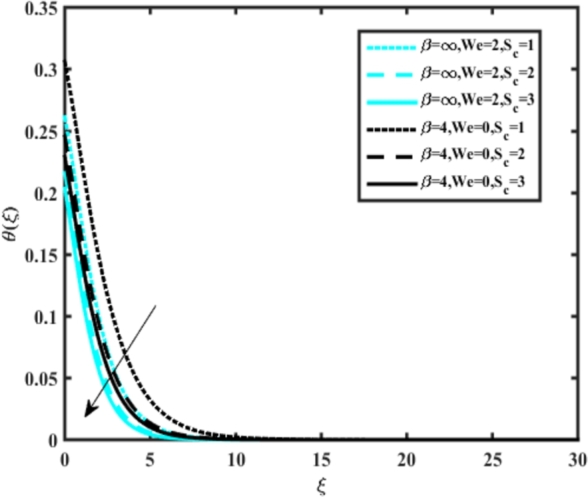
Occurrences of *θ*(*ξ*) for *S*_*c*_ embedded with CCNF.

Fig. 7 illustrates the influence of the Biot number $B_{i1}$ on the temperature profiles of the Casson-Carreau fluid, respectively. Newtonian heating characterizes the heating of a solid surface when in contact with a flowing fluid. It's a common model for scenarios where energy transfers uniformly from the solid surface to the adjacent fluid. The effect of Newtonian heating on temperature profiles stems from heat transfer between the solid and the fluid. In this condition, the solid surface maintains a constant temperature higher than that of the fluid. Heat transfer follows Fourier's law, driven by the temperature difference between the solid and the fluid. As Newtonian heating increases, so does the energy difference between the solid surface and the fluid. This heightened energy gap leads to a steeper temperature gradient at the solid-fluid interface. The temperature velocity distribution of both fluids increases as we increase the pertinent parameter. However, the velocity fluctuation in the Casson model is higher compared to the Carreau model. Similar trends are observed for the Darcy parameter, as seen from Fig. 8. The temperature profile in porous media can rise with increasing values of the porosity parameter due to enhanced convection induced by fluid flow. As porosity increases, more fluid can flow through the medium, leading to enhanced convective heat transport, effectively carrying heat and resulting in a temperature rise within the porous medium. Figure 9, Figure 10 depict variations in the profiles of the Casson-Carreau model with the involvement of parameters $E_{c}$ and mixed bio-convection parameter Ω The Eckert number $E_{c}$ quantifies the balance between kinetic energy (flow velocity) and internal energy (thermal energy). A higher $E_{c}$ indicates a greater proportion of kinetic energy relative to internal energy, enabling the fluid to pick up thermal energy more effectively through convection as it flows through regions with a temperature gradient. Here, again, the temperature velocities are enhanced with larger values of both parameters. Fig. 11 presents the influence of the magnetic parameter *M* on temperature profiles of the Casson-Carreau fluid. An increase in *M* value enhances the temperature field for both fluid phases. The counteracting force introduced as Lorentz drag enhances the generation of frictional heating between the fluid layers, resulting in the production of heat energy and thickening of the thermal boundary layer for both Casson and Carreau fluids, respectively. When discussing Joule heating effects, which are intricately linked with magnetic field intensity, we observe temperature profiles influenced by MHD. As the magnetic field intensity increases, the impact of MHD on temperature profiles becomes more pronounced. Joule heating phenomena occur when electric currents disperse energy as heat through a conductive medium. In the context of MHD, induced electric currents play a significant role, particularly when the flow passes through highly conductive fluids such as plasmas. These induced currents encounter resistance within the fluid, generating heat that contributes to a rise in temperature. Furthermore, higher induced currents strengthen the induced magnetic field, intensifying the Joule heating process and leading to a notable increase in the temperature of the conducting fluid. The decreasing behavior is observed as we enhance the parameter n for both fluid models, as noticed from Fig. 12. Figure 13, Figure 14 show that as the values of $N_{b}$ and *S* increase, the temperature profiles of both Casson-Carreau fluid phases increase. Figure 15, Figure 16 elucidate the influence of the Prandtl number and Schmidt number $S_{c}$ on the temperature profiles of both Casson and Carreau fluids. As the Prandtl number and $S_{c}$ increase, the temperature profiles of both phases exhibit a gradual decline. This phenomenon arises because higher Prandtl numbers indicate lower thermal conductivity in the fluid, leading to reduced heat conduction and thinner thermal boundary layers. Consequently, this results in an increased rate of heat transfer at the surface. An increase in the Prandtl number signifies that thermal diffusivity becomes relatively more dominant compared to kinematic viscosity in fluid flow scenarios. Consequently, the velocity of the fluid surpasses the temperature profile, indicating that heat transfer is less efficient than momentum transfer. Conversely, a decrease in the Prandtl number implies that the fluid exhibits lower kinematic viscosity relative to thermal diffusivity. In such cases, the thermal profile exhibits a higher rate of change compared to the velocity profile, suggesting that heat transfer occurs more vigorously than momentum transfer. When the Prandtl number rises, thermal diffusivity becomes relatively slower compared to kinematic viscosity. Consequently, heat disperses less efficiently through the fluid flow. Moreover, in fluid flow over surfaces, an increase in the Prandtl number leads to a slower thermal diffusivity, resulting in steeper temperature gradients (changes in temperature with respect to distance). This implies that the temperature variation within the fluid is concentrated over a smaller spatial distance.

### 8.3 Graphical discussion of concentration profile for novel parameters

**Figure 17:**
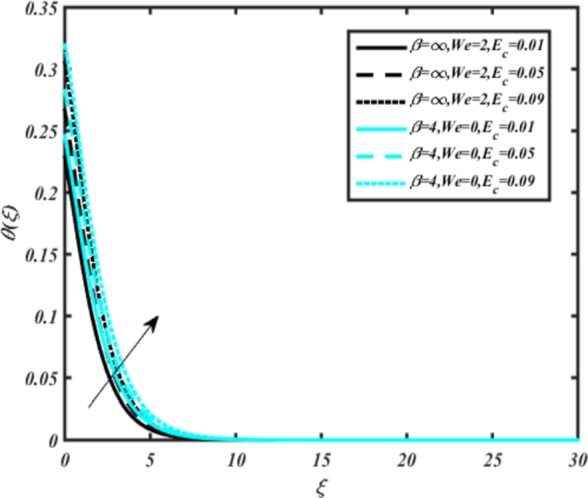
Occurrences of *χ*(*ξ*) for *E*_*c*_ embedded with CCNF.

**Figure 18:**
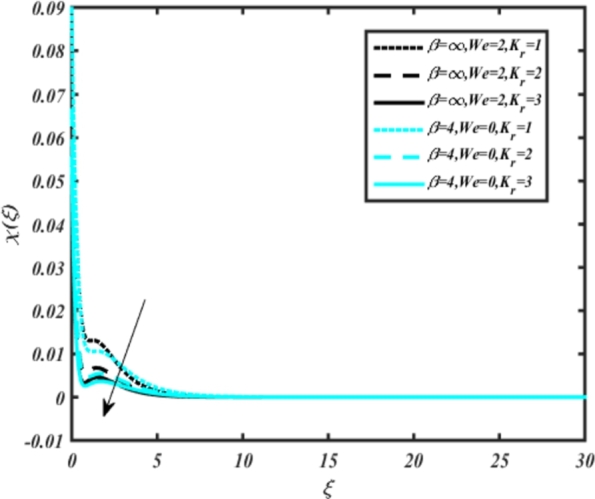
Occurrences of *χ*(*ξ*) for *K*_*r*_ embedded CCNF.

**Figure 19:**
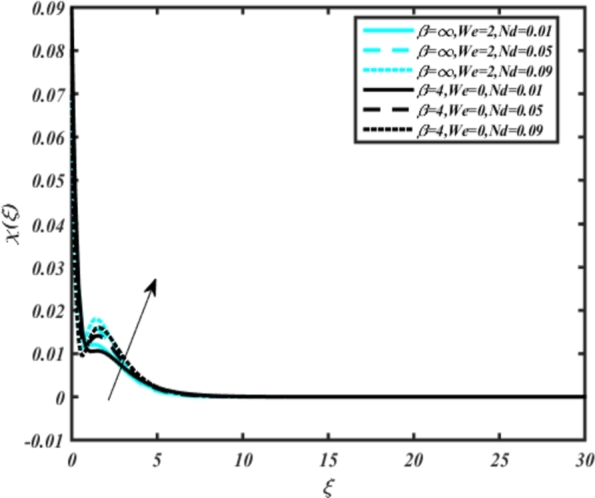
Occurrences of *χ*(*ξ*) for *Nd* embedded with CCF.

**Figure 20:**
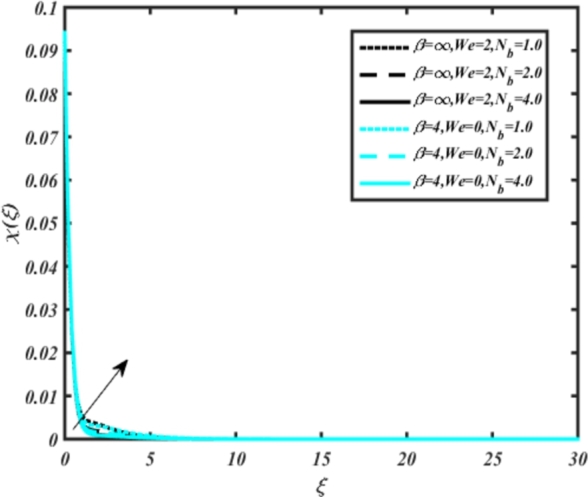
Occurrences of *χ*(*ξ*) for *N*_*b*_ embedded with CCNF.

**Figure 21:**
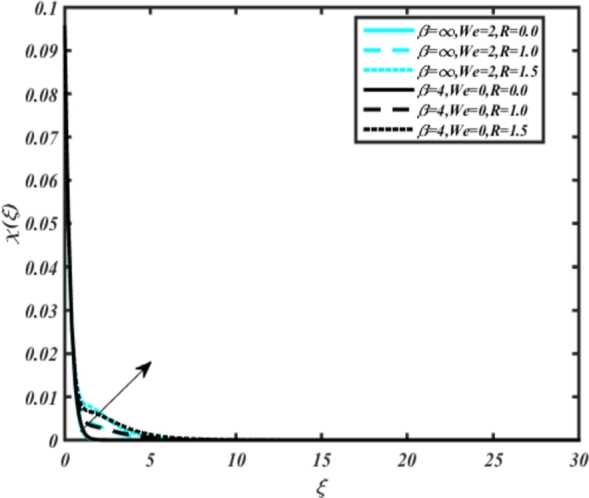
Occurrences of *χ*(*ξ*) for *R* embedded with CCNF.

**Figure 22:**
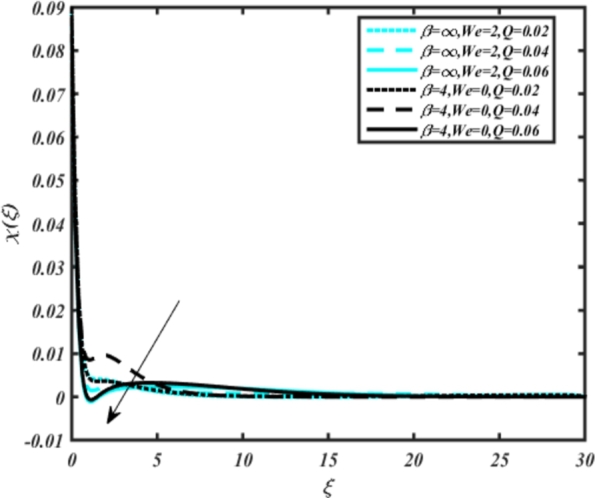
Occurrences of *χ*(*ξ*) for *Q* embedded with CCNF.

**Figure 23:**
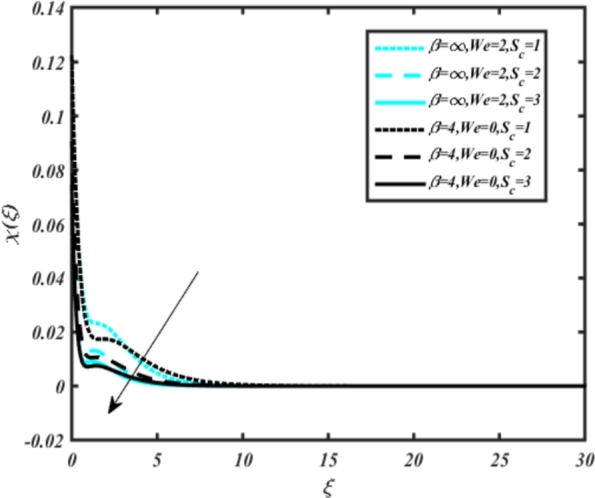
Occurrences of *χ*(*ξ*) for *S*_*c*_ embedded with CCNF.

Figure 17, Figure 18 compare the influence of the Eckert number $E_{c}$ and $K_{r}$ for varying parameters. We observed that an increase in both parameters enhances the concentration profiles of both the Casson and Carreau fluid phases. Chemical reactions within a fluid flow involve the conversion of reactants into products, with reactions occurring between different chemical species. These reactions can either consume one or more species (consumption reactions) or produce new species (production reactions). Consumption reactions decrease reactant concentrations, while production reactions increase product concentrations. When present in a fluid flow, chemical reactions significantly affect concentration profiles. In consumption reactions, reactants are consumed, leading to decreased concentrations near reaction sites. Conversely, production reactions lead to increased concentrations of products, resulting in higher mass near reaction sites. Overall, chemical reactions modify fluid concentration gradients. Figure 19, Figure 20, Figure 21 illustrate the effect of *Nd*, $N_{b}$, and *R* on the temperature velocity of both the Casson-Carreau model. Temperature velocity increases with the values of these parameters. Higher values of the radiation parameter *R* result in the expansion of the thermal boundary layer thickness and increased heat influx into the fluid, enhancing temperature profiles for both the Casson-Carreau fluid and the dust phase. The impetus behind temperature profile alterations due to radioactivity primarily stems from radiative energy transfer, a crucial process for exchanging thermal energy between surfaces or within a medium through electromagnetic waves (radiation). Radiative heat transfer becomes significant when substantial temperature disparities exist and when a material allows radiation to pass through to some extent. In systems where radiation serves as the predominant mode of energy transfer, it manifests through the emission, absorption, and transmission of electromagnetic waves, typically within the infrared spectrum. Thermal radiation emanates from all substances with a temperature above absolute zero. The effect of heat source/sink and the Schmidt number $S_{c}$ on the Casson-Carreau fluid phase is depicted in Figure 22, Figure 23. The concentration profile of both Casson-Carreau fluid phases diminishes with increasing values of these parameters.

### 8.4 Graphical discussion of microbial profile for novel parameters

**Figure 24:**
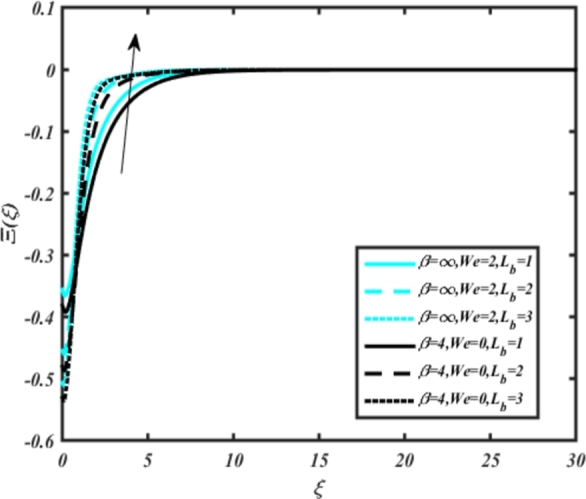
Occurrences of Ξ(*ξ*) for *L*_*b*_ with CCNF.

**Figure 25:**
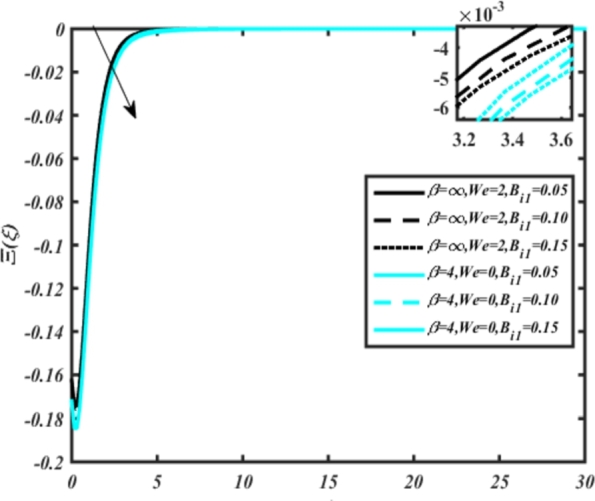
Occurrences of Ξ(*ξ*) for *B*_*i*1_ with CCNF.

**Figure 26:**
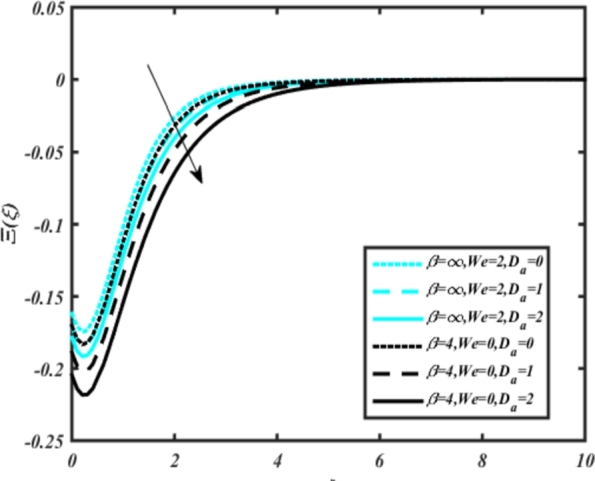
Occurrences of Ξ(*ξ*) for *D*_*a*_ embedded with CCNF.

**Figure 27:**
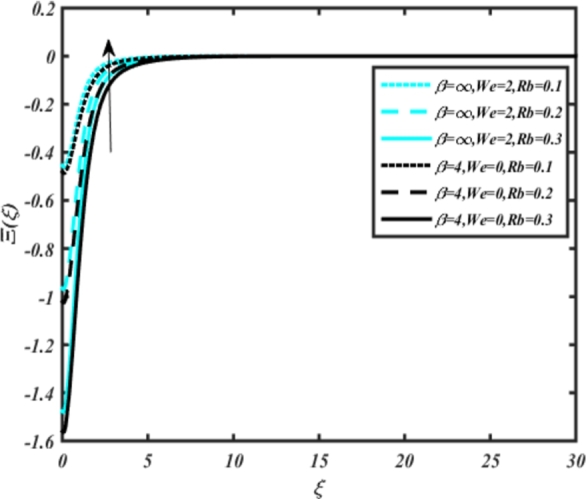
Occurrences of Ξ(*ξ*) for *Rb* embedded with CCNF.

**Figure 28:**
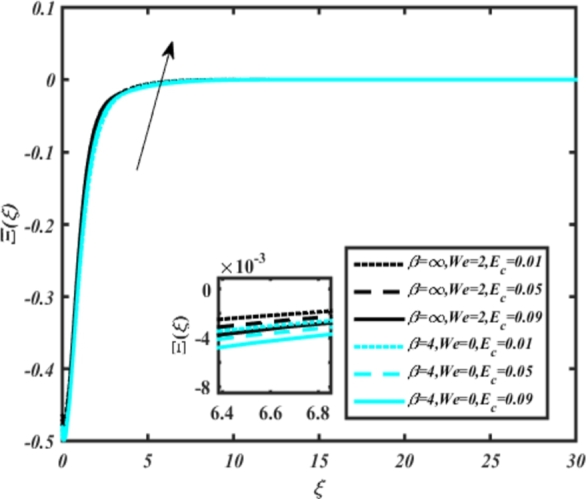
Occurrences of Ξ(*ξ*) for *E*_*c*_ embedded with CCNF.

**Figure 29:**
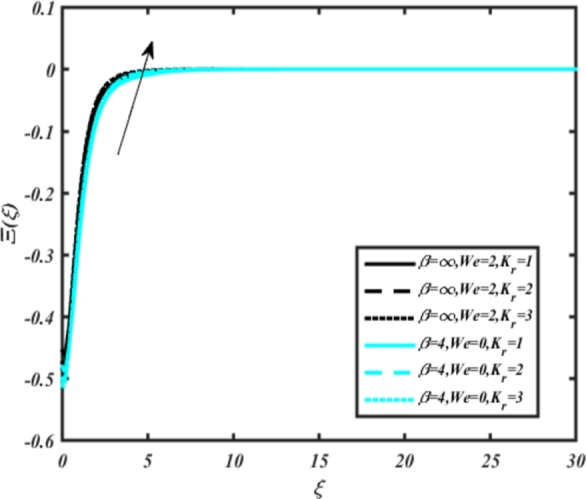
Occurrences of Ξ(*ξ*) for *K*_*r*_ embedded with CCNF.

**Figure 30:**
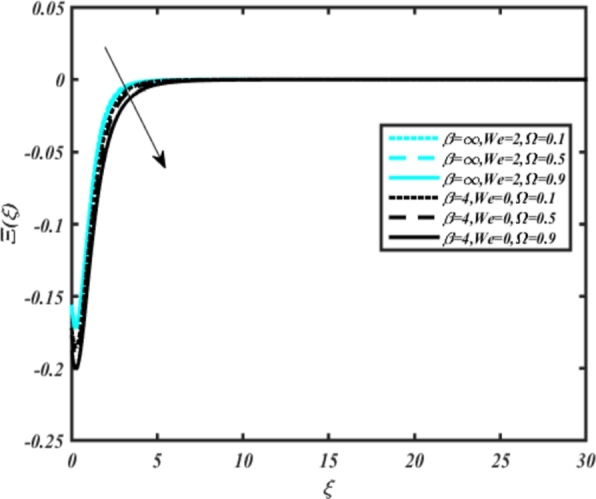
Occurrences of Ξ(*ξ*) for Ω embedded with CCF.

**Figure 31:**
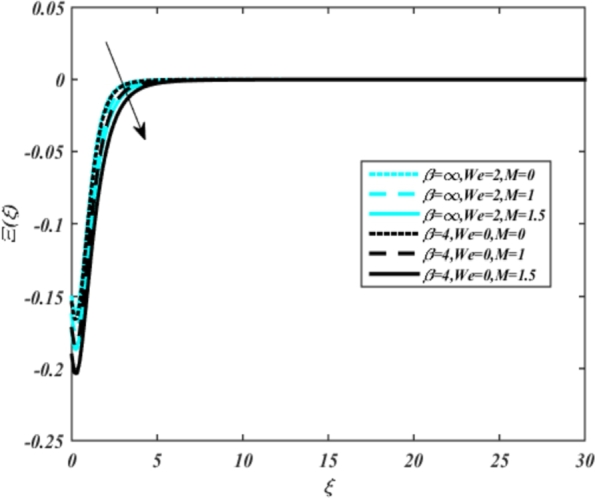
Occurrences of Ξ(*ξ*) for *M* embedded with CCNF.

**Figure 32:**
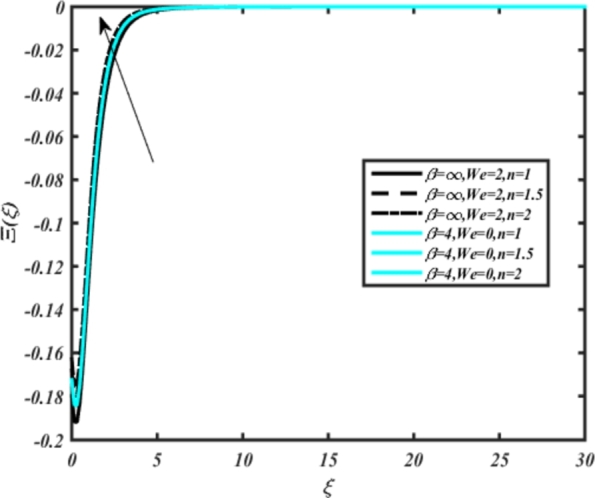
Occurrences of Ξ(*ξ*) for *n* embedded with CCNF.

**Figure 33:**
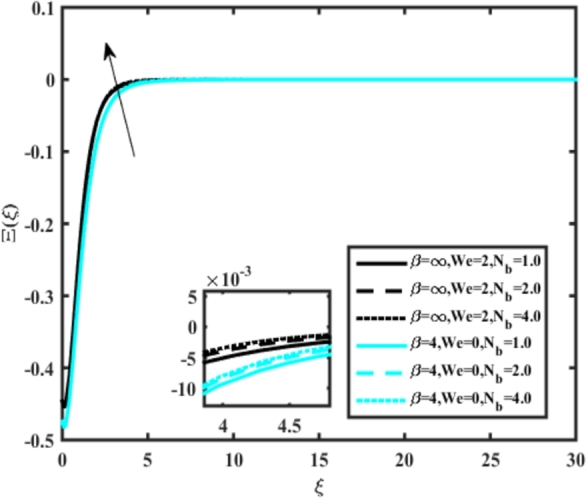
Occurrences of Ξ(*ξ*) for *N*_*b*_ embedded with CCNF.

**Figure 34:**
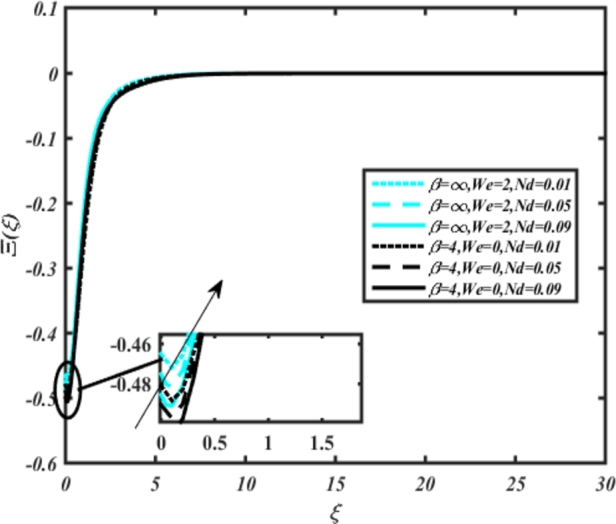
Occurrences of Ξ(*ξ*) for *N*_*d*_ embedded with CCNF.

**Figure 35:**
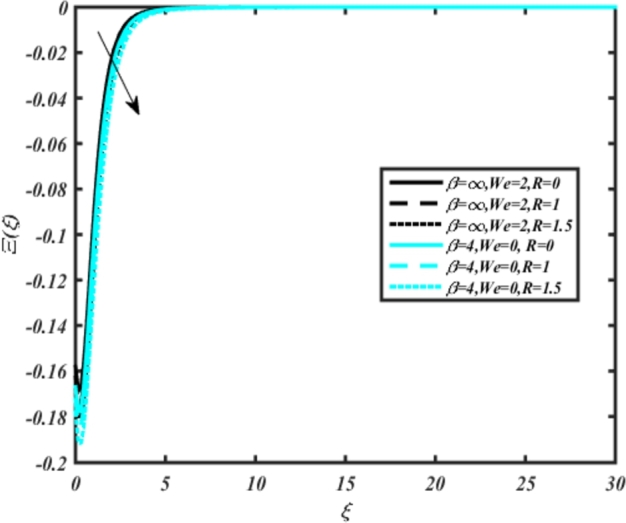
Occurrences of Ξ(*ξ*) for *R* embedded with CCNF.

**Figure 36:**
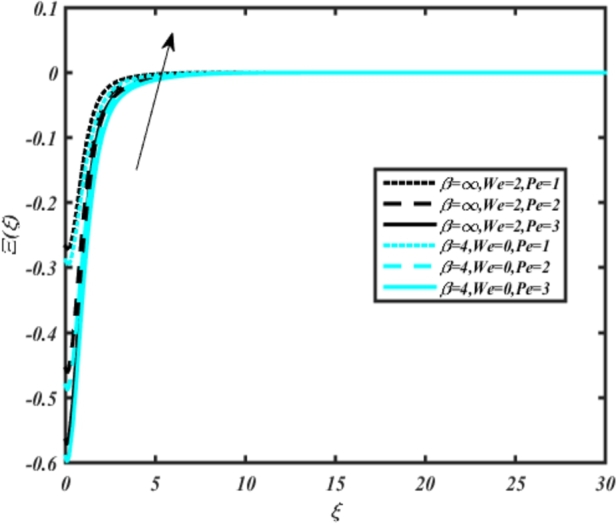
Occurrences of Ξ(*ξ*) for *Pe* embedded with CCNF.

Figure 24, Figure 25, Figure 26, Figure 27, Figure 28, Figure 29, Figure 30, Figure 31, Figure 32, Figure 33, Figure 34, Figure 35, Figure 36 illustrate the influence of various parameters on microbial velocity. In Fig. 24, microbial distributions for different values of are plotted. It is observed that increasing $L_{b}$ values lead to higher temperature distributions in the flow region for both Casson and Carreau fluids. Figure 25, Figure 26 depict the effects of $B_{i1}$ and $D_{a}$ respectively. These plots reveal that microbial velocity decreases as $B_{i1}$ and $D_{a}$ values increase. Figure 27, Figure 28, Figure 29 demonstrate the impact of *Rb*, $E_{c}$, and $K_{r}$ parameters on microbial distribution of CCNF. It is elucidated that microbial distribution increases with increasing values of these parameters. Similarly, Figure 29, Figure 30 illustrate the influence of mixed convection parameter Ω and magnetic parameter *M*. Microbial velocity decreases with the upsurge in the values of both these parameters. The effects of *n*, $N_{b}$, and *Nd* are shown in Figure 32, Figure 33, Figure 34 respectively. Higher values of all these parameters enhance microbial velocity distribution. Lastly, the impact of radiation parameter *R* and Peclet number *Pe* on microbial velocity is depicted in Figure 35, Figure 36. It is observed that microbial velocity decreases with increasing *R* values, while the opposite trend is observed in Fig. 36 for the Peclet number *Pe*.

## 9 Conclusion

In conclusion, this research introduces a comprehensive mathematical model to investigate the flow of a non-Newtonian magneto CCNF over a stretching porous surface, incorporating various transport phenomena such as mass and heat transfer rates, Stefan blowing, non-linear thermal radiation, heat source-sink effects, chemical reactions, thermophoretic and Brownian motions, convective heating, Joule heating, and the presence of motile microorganisms leading to bio-convection. By employing microorganisms, the suspension of nanomaterials within the nanofluid is effectively controlled. The boundary layer approximation yields highly nonlinear PDEs, which are then simplified using the novel Lie group theoretical method and the one-parameter Lie scaling method, thereby converting them into ODEs. The numerical solutions for these ODEs are obtained using the bvp4c scheme built-in function in MATLAB, ensuring reliable outcomes for temperature, velocity, concentration, and motile microorganism density profiles. The comparison of numerical results with available data demonstrates good agreement, highlighting the validity of the proposed model. Overall, the numerical outcomes provide valuable insights into various flow characteristics and phenomena within the studied system. The few key points about the results are:

1. In Table 1, we observe that the impact of the Bioconvection Rayleigh number is positive in both the Casson fluid and Carreau fluid models, with the Casson fluid model exhibiting a notably higher rate of change. On the other hand, the buoyancy ratio parameter shows consistent rates of change across both models, while the mixed bioconvection parameter has a more pronounced reduction in impact for the Casson fluid model compared to the Carreau fluid model.

2. For instance, $B_{i1}$ shows a significant decrease in the Nusselt number, accompanied by an increase in the Sherwood number and motile density number. Conversely, $B_{i2}$ demonstrates an increase in the Sherwood number but a decrease in the other two quantities. Lastly, $B_{i3}$ exhibits a notable rate of change in the motile density number, indicating rapid decay, while the other quantities show slower growth.

3. Increasing the magnetic parameter *M* leads to decreased velocity profiles for both Carreau (blue) and Casson (black) fluids, as illustrated in Fig. 3.

4. Fig. 4 demonstrates that larger *Q* values result in increased velocity for both Carreau (blue) and Casson (black) fluids.

5. Fig. 5 illustrates that the mixed bio-convection parameter Ω decreases momentum velocity for both Carreau and Casson fluids. Similarly, Fig. 6 depicts how the Darcy parameter $D_{a}$ enhances fluid velocity for both Carreau and Casson fluids.

6. Larger values of Newtonian heating and Darcy parameter enhance temperature velocities, with higher fluctuations observed in the Casson model compared to the Carreau model.

7. Increasing values of the porosity parameter and mixed bio-convection parameter result in enhanced temperature profiles for both Casson and Carreau fluids.

8. Higher values of the Eckert number and magnetic parameter lead to increased temperature fields, with the magnetic parameter introducing Lorentz drag and enhancing frictional heating.

9. Increasing the power law index, Schmidt number, and Prandtl number leads to a decrease in temperature profiles for both fluid phases.

10. Figure 17, Figure 18 show that increasing the Eckert number $E_{c}$ and parameter $K_{r}$ enhances concentration profiles for both Casson and Carreau fluid phases.

11. Figure 19, Figure 20, Figure 21 demonstrate that temperature velocity increases with parameters *Nd*, $N_{b}$, and *R*, respectively, for the Casson-Carreau model.

12. Higher radiation parameter *R* values expand the thermal boundary layer and increase heat influx into the fluid, enhancing temperature profiles.

13. Figure 22, Figure 23 show the effect of heat source/sink and Schmidt number on concentration profiles of the Casson-Carreau fluid phase, revealing that increasing these parameters diminishes concentration profiles.

14. Increasing values of the parameter $L_{b}$ result in higher microbial velocity distributions in the flow region for both Casson and Carreau fluids (Fig. 24).

15. Microbial velocity decreases as the $B_{i1}$ and $D_{a}$ values increase, as shown in Figure 25, Figure 26, respectively.

**Limitations:**

1. The flow model is constrained to Casson-Carreau fluid models.

2. Assumption of linear stretching porous plate.

3. Consideration of solely similar solutions, restricting the exploration of non-similar flow behaviors.

4. Focus limited to one-phase fluid flow phenomena.

5. Absence of viscous dissipation in the analysis, potentially restricting the representation of certain physical effects.

6. Exclusive reliance on the bvp4c numerical technique.

7. The conversion of PDEs to ODEs is accomplished through the one-parameter scaling method.

**Future Directions:**

1. Expand the study to incorporate the Upper Convected Maxwell fluid model for a more comprehensive representation of fluid behavior.

2. Investigate non-linear stretching surface configurations to broaden the analysis scope.

3. Explore linear shrinking surface cases, conducting dual simulations to assess stability and potentially uncover multiple solutions.

4. Include dusty fluid flow phases to address additional complexities in the system.

5. Enhance accuracy and efficiency by replacing the numerical method with alternatives such as RK-4, RK-Felberg, or spectral methods, along with the Keller box method.

6. Utilize similarity transformations or the similar considered method after selecting the unsteady frame to further explore the problem.

**Real world applications:**

The studied flow phenomena have wide-ranging applications across various research domains, industrial processes, laboratory settings, and medical fields.

1. In biomedical engineering, microfluidic devices are integral to drug delivery systems. Utilizing the CCNF model allows for the design and optimization of these devices, ensuring precise drug delivery. The controlled thermal environment within these devices, influenced by factors such as nanoparticles movement and chemical reactions, plays a crucial role in regulating drug release rates. Incorporating bioconvection phenomena and heat source-sink dynamics enhances the accuracy of drug delivery predictions, making these devices indispensable in biomedical applications.

2. Specifically tailored flow phenomena and mathematical models find utility in hyperthermia treatment within the medical field. Microfluidic devices are employed to induce specific flow patterns and control temperature profiles, facilitating targeted heating of affected tissues. By leveraging an understanding of bioconvection and heat dynamics, these devices optimize therapeutic outcomes in hyperthermia treatment, ensuring effective and precise tissue heating for improved patient outcomes.

3. Industrial engineering benefits from mathematical models based on flow phenomena, particularly in processes such as dyeing fiber sheets. Understanding flow patterns and thermal characteristics is essential for achieving uniform coloration in fiber sheets during the dyeing process. By applying similar mathematical models used in biomedical engineering, industrial engineers can optimize heating processes, ensuring consistent and high-quality results in fiber sheet dyeing operations.

## CRediT authorship contribution statement

**Musharafa Saleem:** Writing – review & editing, Software, Methodology, Conceptualization. **Majid Hussain:** Writing – review & editing, Writing – original draft, Supervision, Investigation, Conceptualization.

## Declaration of Competing Interest

The authors declare the following financial interests/personal relationships which may be considered as potential competing interests: Musharafa Saleem reports writing assistance was provided by University of Management and Technology. Musharafa Saleem reports a relationship with University of Management and Technology that includes: employment. If there are other authors, they declare that they have no known competing financial interests or personal relationships that could have appeared to influence the work reported in this paper.
